# *Mycobacterium tuberculosis* Rv1987 protein attenuates inflammatory response and consequently alters microbiota in mouse lung

**DOI:** 10.3389/fcimb.2023.1256866

**Published:** 2023-11-01

**Authors:** Yingying Liu, Jiaqi Zhang, Guangxian Leng, Junxing Hu, Wenzhen Wang, Guoying Deng, Yufang Ma, Shanshan Sha

**Affiliations:** ^1^ Department of Biochemistry and Molecular Biology, Dalian Medical University, Dalian, Liaoning, China; ^2^ Department of Microbiology, Dalian Medical University, Dalian, Liaoning, China

**Keywords:** *Mycobacterium tuberculosis*, Rv1987, lung microbiota, immune response, metabolites

## Abstract

**Introduction:**

Healthy lung microbiota plays an important role in preventing *Mycobacterium tuberculosis* (Mtb) infections by activating immune cells and stimulating production of T-helper cell type 1 cytokines. The dynamic stability of lung microbiota relies mostly on lung homeostasis. In our previous studies, we found that Mtb virulence factor, Rv1987 protein, can mediate host immune response and enhance mycobacterial survival in host lung. However, the alteration of lung microbiota and the contribution of lung microbiota dysbiosis to mycobacterial evasion in this process are not clear so far.

**Methods:**

*M. smegmatis* which does not contain the ortholog of Rv1987 protein was selected as a model strain to study the effects of Rv1987 on host lung microbiota. The lung microbiota, immune state and metabolites of mice infected by *M. smegmatis* overexpressing Rv1987 protein (MS1987) were detected and analyzed.

**Results:**

The results showed that Rv1987 inhibited inflammatory response in mouse lung and anaerobic bacteria and *Proteobacteria*, *Bacteroidota*, *Actinobacteriota* and *Acidobacteriota* bacteria were enriched in the lung tissues correspondingly. The immune alterations and microbiota dysbiosis affected host metabolic profiles, and some of significantly altered bacteria in MS1987-infected mouse lung, such as *Delftia acidovorans*, *Ralstonia pickettii* and *Escherichia coli*, led to anti-inflammatory responses in mouse lung. The secretory metabolites of these altered bacteria also influenced mycobacterial growth and biofilm formation directly.

**Conclusion:**

All these results suggested that Rv1987 can attenuate inflammatory response and alter microbiota in the lung, which in turn facilitates mycobacterial survival in the host.

## Introduction

Tuberculosis (TB) threads human health worldwide especially in developing countries. In 2021, there were 6.4 million new diagnosed TB cases and 1.6 million people were dead from TB (World Health Organization, 2022). More comprehensive understanding of *Mycobacterium tuberculosis* (Mtb) infection, including the key bacterial components, the immune state of host lung and other factors that influence TB development, will be great significance for the development of new anti-tuberculosis drugs.

Lung microbiota and its dysbiosis have been reported to be closely associated with various lung diseases including TB in recent years. Previously, the lung was believed to be free from bacteria; however, with the development of the culture-independent techniques that directly amplify and analyze the 16S rRNA of bacteria, the lung is revealed to be not sterile but colonized by diverse communities of microbiotas (Dickson et al., 2016; Man et al., 2017). At the phylum level, the most common bacteria in human lungs are Bacteroidetes, Firmicutes, and Proteobacteria, and in genus level, the dominant bacteria are Prevotella, Veillonella, Pseudomonas, Fusobacteria, and Streptococcus (Beck et al., 2012). Mice have similar bacterial composition in the lung to humans (Barfod et al., 2013). These bacteria probably come from upper respiratory tract and keep a balance between the immigration from upper respiratory tract and the elimination from lung (Charlson et al., 2011). In the lung, these bacteria maintain immune homeostasis and active host defense against the infection of pathogenic bacteria and viruses by regulating macrophages functions (Bernasconi et al., 2016), stimulating Th1 cytokines secretion (Shenoy et al., 2017), inducing Th17 lymphocyte differentiations (Segal et al., 2016) and promoting neutrophil activation (Horn et al., 2022). In TB patients, the lung microbiota profiles are significantly different from healthy individuals (Zhou et al., 2015; Vázquez-Pérez et al., 2020; Xiao et al., 2022). The diversity of lung microbiota is diminished in TB patients. However, the reasons causing lung microbiota alterations in TB patients and the effects of microbiota dysbiosis on TB development are still not clarified.

Immune homeostasis is the key factor that influences the microbiota composition in local tissues. The studies on LPS-induced lung injury and HIV patients prove that any alteration in immune response will change the diversity and the structure of bacteria in the lung (Poroyko et al., 2015; Twigg et al., 2016). Therefore, we speculate that the key Mtb effector molecules regulating host immune will have impacts on microbiota composition. Rv1987 is a secretory protein (de Souza et al., 2011) encoded by *rv1987* gene which locates in virulence-related RD2 region of Mtb genome (Parkash et al., 2009). The orthologs of Rv1987 protein exist in most pathogenic mycobacterium species, but not in non-pathogenic mycobacterium, such as *Mycobacterium smegmatis* (Mba Medie et al., 2010). Rv1987 is predicted to be a chitinase and has been reported to have cellulose-binding activity (Mba Medie et al., 2011). In our previous studies, we found that Rv1987 protein induced Th2 response in mice systemically (Sha et al., 2017) and promoted M2-like polarization of alveolar macrophages in mouse lung (Sha et al., 2021). The *M. smegmatis* strain overexpressing Rv1987 protein (MS1987) had an enhanced survival ability in mice compared to *M. smegmatis* carrying empty vector (MSVec) (Sha et al., 2017). However, the alteration of lung microbiota and the contribution of lung microbiota dysbiosis to mycobacterial evasion in this process are not clear so far.

In this study, MS1987 and MSVec strains were used to infect C57BL/6 mice and the effects of Rv1987 protein on the local immune state of the lung were firstly studied. The resulting changes in lung microbiota of MS1987-infected mice compared to MSVec-infected mice were analyzed by both 16S rRNA sequencing and *in vitro* cultivation. Furthermore, the impacts of lung microbiota dysbiosis on metabolic microenvironment and immune hemostasis of the lung were observed. All the results will be helpful for understanding how Mtb employs its virulence factors to alter lung microbiota and consequently promotes its own survival in the host lung.

## Materials and methods

### Bacteria and mice

An *M. smegmatis* strain overexpressing Rv1987 protein (MS1987) and an *M. smegmatis* strain carrying the empty vector (MSVec) were constructed in our previous study (Sha et al., 2017). They were grown in LBT broth and LB agar plates containing 25 μg/mL kanamycin. Aerobic bacteria *Delftia acidovorans* and *Ralstonia pickettii* were obtained from BeNa Culture Collection (Zhengzhou, Henan province, China). Anaerobic bacterium *Escherichia coli* AW1.7 was isolated from mouse lung in this study. All these bacteria were confirmed by 16S rRNA sequencing before the infection experiments. *E. coli* AW1.7 was cultured in Glfu Anaerobic Medium (GAM) at 37°C under anaerobic condition. *D. acidovorans* and *R. pickettii* were grown in nutrient medium (3 g beef extract, 10 g peptone and 5 g NaCl in 1 L water) at 37°C. Specific, pathogen-free female (SPF) C57BL/6 mice of 6 to 8 weeks old were bred and maintained in the SPF animal facility at Laboratory Animal Center of Dalian Medical University, China. After infection by bacteria, the mice were maintained in a clean environment illuminated for 12 hours each day and kept at 24 ± 1°C with free access to sterilized food and water.

### Infection of mice with bacteria

To study the effect of Rv1987 protein on the microbiota and immune alterations in host lung, C57BL/6 mice were challenged with MS1987 or MSVec by inhalation. To study the influence of altered bacteria in MS1987-infected mouse lung (*E. coli*, *D. acidovorans*, or *R. pickettii*) on the inflammatory responses in host lung, C57BL/6 mice were infected with these three bacteria independently by inhalation. The relationship between the optical density at 600 nm (OD_600_) of bacterial culture and colony forming unit (CFU) values were determined firstly. As for anaerobic bacterium *E. coli* AW1.7, the growth rates of bacteria exposed to aerobic conditions for different time were determined before infection. The bacilli suspended in PBS buffer were aerosolized using aerosol generator (Kangjie Instrument, China) and delivered to each animal at 5 × 10^9^ CFU/day for MS1987 and MSVec, or at 5 × 10^8^ CFU/day for *D. acidovorans*, *R. pickettii*, and *E. coli*, and the infection lasted 4 days. The mice without infection were set as controls (CN). Five to seven mice were assigned in one group. At day 9 or day 16 after the first day of infection, the mice were sacrificed by spinal cord dislocation. The blood was taken from eyes and centrifuged at 500 x g for 10 min. The sera were separated and stored at -80°C. The lung tissues were collected sterilely, washed with PBS, and divided into several parts rapidly. The lung tissues for RNA isolation and protein preparation were stored at -80°C. The lung tissues for metabolite analysis were rapidly immerged in liquid nitrogen for 10 min and then stored at -80°C. The lung tissues for histopathology were immerged in 10% formalin in PBS. The lung tissues for bacterial CFU counting, culturation and DNA isolation were operated immediately.

For re-infection experiments, the mice were infected firstly by MS1987 or MSVec as described above. Then, at day 9 post-primary-infection, each mouse was infected by MSVec at 5 × 10^9^ CFU/day for 2 days. The lung tissues were collected for CFU counting at day 2 post-secondary-infection.

### RNA isolation and RT-qPCR

The total RNA in mouse lung tissues was isolated with the RNAiso plus reagent (TaKaRa, Otsu, Japan) according to the manufacturer’s instructions. The quantity of RNA was determined by reading its absorbance at 260 nm. Then, 1 μg of isolated RNA was immediately reverse transcribed to cDNA using the PrimeScriptTM RT reagent kit with gDNA Eraser (TaKaRa). The cytokines TNF-α, IL-12 *p40*, IL-17, IL-4, IL-10, and IFN-γ were determined by qPCR with the TB Green^®^ Premix Ex Taq™ II kit (TaKaRa). The primers were shown in **Supplementary Table 1**.

### ELISA

The expression of secretory cytokines IL-17 and IL-10 in sera and lung tissues was determined using ELISA kits (Abcam, Cambridge, UK) according to the manufacturer’s instructions. For sera, 10 μL of mouse sera was applied in the microwells coated by capture antibodies. For lung tissues, the protein solution was prepared for ELISA detection firstly. Twenty mg of lung tissues were homogenized in 0.2 mL cold PBS buffer with 10 mM phenylmethylsulfonyl fluoride (PMSF). After centrifugation at 10000 x g for 10 min, the supernatant containing proteins was transferred to another tube and 10 μL of this solution was added in microwells. After sample application, the microwells were added by detection antibody conjugated with horse-radish peroxidase (HRP) and finally developed colors with HRP substrates which were read at 450 nm. The concentration of cytokine in each sample was calculated by the standard curve.

### Bacterial culture and colonies analysis

Fifty milligram of mouse lung tissues were mechanically homogenized in 1 mL sterile PBS buffer. Forty microliters of the homogenate from each mouse of one group were mixed together. Half of the mixture was injected into the aerobic blood enrichment culture, and the other half was injected into the anaerobic blood enrichment culture (Scenker, Liaocheng, China). The bacteria were cultured in aerobic and anaerobic condition at 37°C for 5 days respectively. Then, 10 μL of the culture was serially diluted and finally spread on tryptic soy agar (TSA) plates, blood agar base (BAB) plates and De Man, Rogosa, and Sharpe (MRS) agar plates. The colonies that were different in numbers between MS1987 and MSVec groups were cultured in corresponding liquid medium. The bacterial genomic DNA were isolated with TIANamp bacteria DNA kit (Tiangen Company, Beijing, China). The V3-V8 region of bacterial 16S rRNA was amplified by PCR with the primers: 27F: AGAGTTTGATCMTGGCTCAG and 1492R: GGTTACCTTGTTACGACTT. The PCR products were then sequenced and compared with the sequences in GenBank database to identify the bacterial species.

### 16S rRNA gene amplicon sequencing and analysis

The bacterial DNA for 16S rRNA sequencing was isolated immediately after the lung tissues were collected from mice. The remaining homogenate of mouse lung tissues after bacterial culture experiment was centrifuged at 14000 x g for 10 min. The pellet was collected for isolating the bacterial DNA using fecal DNA extracting kit (Tiangen) according to the manufacturer’s instructions. The concentration of isolated DNA was determined by reading its absorbance at 260 nm. Then, the V3-V4 region of 16S rRNA was amplified by PCR with primers 341F: CCTAYGGGRBGCASCAG and 806R: GGACTACNNGGGTATCTAAT. The reactions contained 15 μL of Phusion High-Fidelity PCR Master Mix (New England Biolabs), 1 μM of forward and reverse primers, and 10 ng template DNA. The PCR products were detected by 2% agarose gel electrophoresis and then purified using the Universal DNA Purification Kit (Tiangen). Sequencing libraries were generated using NEB Next Ultra DNA Library Pre Kit (NEB, USA). The sequence was finally analyzed by NovaSeq 6000 (Illumina, San Diego, CA, USA). The identified sequences were then filtered and the effective operational taxonomic units (OTUs) were used to annotate bacterial species, analyze α-diversity and β-diversity, perform principal-coordinate analysis (PCoA) analysis, and determine significantly different species between groups. α-diversity shows the observed species in the samples of each group directly, while β-diversity presents the weighted unifrac values of samples which are calculated according to the abundance and the phylogenetic distance of OTUs (Lozupone et al., 2007; Lozupone et al., 2011). The differences between groups in α-diversity and β-diversity were compared by wilcox multiple comparisons. PCoA analysis was performed with the principal coordinates that had the highest contribution to unifrac values. The top ten abundant bacteria at phylum, class, order, family and species levels in the lung of MS1987-infected mice, MSVec-infected mice and uninfected mice were listed. Their differences in abundance between groups were shown in heatmap.

### Metabolites analysis by LC-MS/MS

Accurate 100 mg of lung tissues were added with 1 mL cold tissue extract (75% 9:1 methanol: chloroform, 25% H_2_O) and 3 steel beads. The tissues were ground for two times by a high-throughput tissue grinder for 60 seconds at 50 Hz. Then they were sonicated at room temperature for 30 min and put on ice for 30 min. After centrifugation at 4°C for 10 min at 12000 x g, 850 μL supernatant of each sample was transferred into a new centrifuge tube and dried by vacuum. The samples were then dissolved in 200 μL 2-chlorobenzalanine (4 ppm) 50% acetonitrile solution, and filtered through 0.22 μm membrane for LC-MS/MS detections. Here, 20 μL from each sample were mixed to form the quality control (QC) samples, which were used to monitor deviations of the analytical results from these pool mixtures and compare them to the errors caused by the analytical instrument itself.

For LC-MS/MS analysis, the samples were separated with an ACQUITY UPLC^®^ HSS T3 (150×2.1 mm, 1.8 μm, Waters) column maintained at 40°C. The temperature of the autosampler was 8°C. Gradient elution of analytes was carried out with 0.1% formic acid in water (C) and 0.1% formic acid in acetonitrile (D) for positive model, or 5 mM ammonium formate in water (A) and acetonitrile (B) for negative model, at a flow rate of 0.25 mL/min. Injection of 2 μL of each sample was done after equilibration. An increasing linear gradient of solvent B (v/v) was used as follows: 0~1 min, 2% B/D; 1~9 min, 2%~50% B/D; 9~12 min, 50%~98% B/D; 12~13.5 min, 98% B/D; 13.5~14 min, 98%~2% B/D; 14~20 min, 2% D positive model (14~17 min, 2% B-negative model). The Electrospray Ionization Tandem Mass Spectrometry (ESI-MSn) experiments were used with the spray voltage of 3.5 kV and -2.5 kV in positive and negative modes, respectively. Sheath gas and auxiliary gas were set at 30 and 10 arbitrary units, respectively. The capillary temperature was 325°C. The Orbitrap analyzer scanned over a mass range of *m/z* 81-1,000 for full scan at a mass resolution of 70,000. The MS raw data was processed by XCMS package of R language to accomplish peak identification, filtration and alignment. The *m/z*, retention time and intensity of precursor molecules in both positive and negative modes were obtained. All peak areas were normalized using the QC sample before further analysis. The normalized data were imported into SIMCA-P (v13.0) and ropls package of R language to perform multivariate analyses. The PLS-DA analysis was carried out to describe the distribution of metabolites in different groups. The parameters of R2Y and Q2 were applied to evaluate the quality of the PLS-DA mode. The significantly different metabolites between MS1987 and MSVec groups (MWs error < 30 ppm, *p* < 0.05) were classified according to their metabolic pathways at *p* < 0.05 via KEGG database (https://www.kegg.jp/).

### Lung histopathology

The lower lobe of right lung was obtained from the infected mice and fixed with 10% formalin in PBS. Pathological slides were prepared and stained using H&E as described in report (Basaraba et al., 2006). The slides were finally observed with CaseViewer 2.4 software (3DHISTECH Ltd. Budapest, Hungary).

### Growth of mycobacterium and its biofilm with secretory metabolites of other bacteria

The fresh grown culture of *D. acidovorans*, *R. pickettii*, *E. coli* AW1.7 and MSVec was filtered through 0.22 μM membrane and the cell-free culture (CFC) of each bacterium were collected. Five hundred microliter of the CFC of *D. acidovorans*, *R. pickettii*, or *E. coli* AW1.7 were added in 4 mL medium containing 10 μL fresh grown MSVec bacteria. The bacteria were then cultured at 37°C by shaking and their OD_600_ and CFU at different time points were determined. As for biofilm growth, 2.5 mL or 150 μL of MSVec with OD_600_ about 0.1 was inoculated in 12- or 96-well plates. Then, 313 μL or 19 μL CFC of *D. acidovorans*, *R. pickettii* or *E. coli* AW1.7 was added in 12- or 96-well plates. The bacteria were grown statically for 4 days and their biofilms were detected by weighing and crystal violet (CV) staining. In these experiments, MSVec grown with its own CFC was set as the control. As for CV staining, the biofilm formed in 96-well was washed by PBS for three times and then stained by 200 μL 0.1% CV reagent for 15 min at room temperature. After washing by PBS, the CV dye binding on the biofilm was dissolved in 200 μL 95% ethanol. The solution was centrifuged for 2 min at 6000 ×g and the OD_595_ of the supernatant was finally read by a microplate reader.

### Bacterial CFU

For determination of bacterial load in mouse lung, 80 mg lung tissues were cut into small pieces and homogenized in 0.5 mL sterile PBS buffer. The homogenates were serially diluted with PBS and plated on LB agar plates containing 25 μg/mL kanamycin and incubated at 37°C. The bacterial CFU was counted after 3 days. For *in vitro* CFU determination, the fresh bacterial culture was serially diluted directly and spotted on the agar plates.

### Statistical analysis

Statistical analyses were performed using GraphPad Prism 6.01 software. For cytokines, one-way ANOVA analysis with *post-hoc* Tukey’s multiple comparisons was used to determine the difference between MSVec, MS1987 and uninfected control (CN) groups. Unpaired *t*-test was carried out to compare the difference of cytokines between *D. acidovorans*, *R. pickettii* or *E. coli* with CN group. For CFU counting, biofilm weight and CV staining, unpaired *t*-test was used to analyze the difference between one experimental group and the control.

## Results

### Rv1987 alters lung microbiota in mice

**Figure 1:**
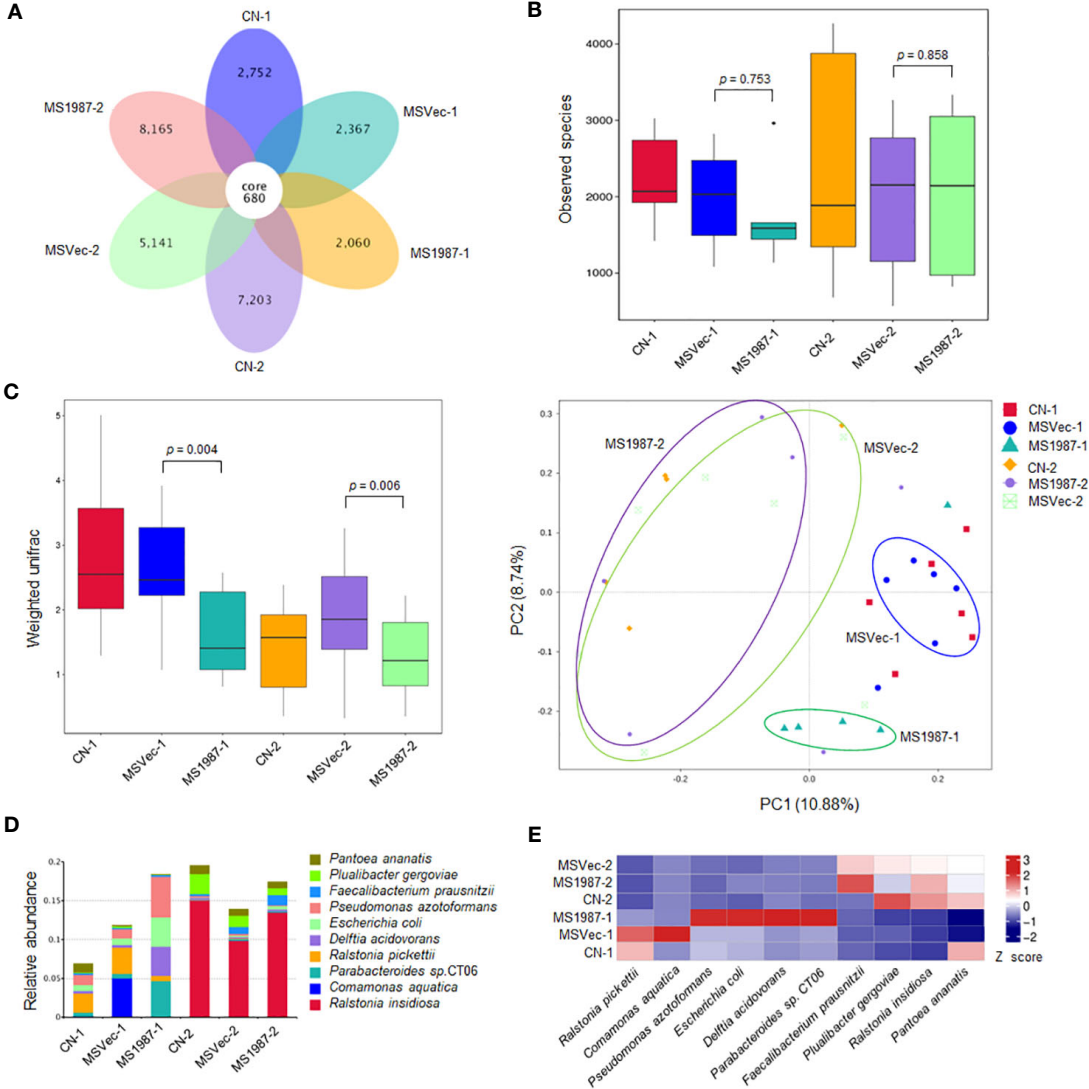
The lung microbiota differs between MS1987- and MSVec-infected mice in 16S rRNA sequencing. The mouse was infected by MS1987 or MSVec at 5 × 10^9^ CFU/day for 4 days and the lung tissues were collected at day 9 and day 16 post-infection. **(A)** The OTUs observed in the lung of MS1987-infected mice, MSVec-infected mice, and uninfected mice. **(B)** α-diversity analysis of the observed species in each group. **(C)** β-diversity and PCoA analyses of the observed species in each group. **(D)** The top ten abundant bacteria at species level in the lung of MS1987-infected mice, MSVec-infected mice and uninfected mice. **(E)** The heatmap of different bacteria in the lung between MS1987- and MSVec-infected mice. The results were from 5-7 mice of each group. MS1987, *M. smegmatis* overexpressing Rv1987 protein; MSVec, *M. smegmatis* carrying empty pVV2 vector; CN, uninfected control. CN-1, MSVec-1 and MS1987-1 represented the samples collected at day 9 post-infection; CN-2, MSVec-2 and MS1987-2 represented the samples collected at day 16 post-infection.

To investigate the effect of Rv1987 protein on lung microbiota composition, the lung microbiota of MS1987- and MSVec-infected mice were analyzed by 16S rRNA sequencing. The results showed that the OTUs identified in MS1987 group was less than that in MSVec group at day 9 post-infection, but was higher than that in MSVec group at day 16 post-infection (**Figure 1A**). The α-diversity analysis revealed that the observed species in MS1987 group was less than that in MSVec group at day 9 post infection, and this difference disappeared at day 16 post-infection when the observed species and their variations were all increased in each group (**Figure 1B**). It was probably because MS1987 and MSVec were almost cleared by host after 16 days post-infection as we reported previously (Sha et al., 2017) and the recovery of lung microbiota had begun. The β-diversity analysis found that the bacterial diversity between MS1987 and MSVec groups was different both at day 9 and day 16 post-infection (**Figure 1C**). The presence of Rv1987 protein led to a decreased bacterial diversity at day 9 post-infection, which may be because the rapid growth of some species in the lung of MS1987-infected mice inhibited the propagation of other bacteria. PCoA analysis revealed that the cluster of MS1987 samples clearly separated from that of MSVec samples at day 9 post-infection (**Figure 1C**), suggesting a divergent composition of lung microbiota between MS1987- and MSVec-infected mice. Further analysis of the top ten abundant bacteria (**Supplementary Figure 1**) showed that at the phylum level, *Proteobacteria*, *Bacteroidota*, *Actinobacteriota* and *Acidobacteriota* were increased in MS1987-infected mouse lung compared to MSVec-infected mouse lung. At the class level, *Alphaproteobacteria* and *Bacteroidia* were elevated in MS1987 group. At the order level, *Enterobacterales*, *Bacteroidales*, *Pseudomonadales* and *Rhizobiales* were enhanced but *Burkholderiales* was reduced in MS1987 group. At the family level, *Enterobacteriaceae* and *Tannerellaceae* were increased, but *Burkholderiaceae* was decreased in MS1987 group. At the species level, *Pseudomonas azotoformans*, *Escherihia coli*, *Delftia acidovorans*, and *Parabacteroides* sp. CT06 were increased in MS1987 group compared to MSVec group, while *Ralstonia pickettii* and *Comamonas aquatica* were decreased significantly at day 9 post-infection (**Figures 1D, E**).

**Figure 2:**
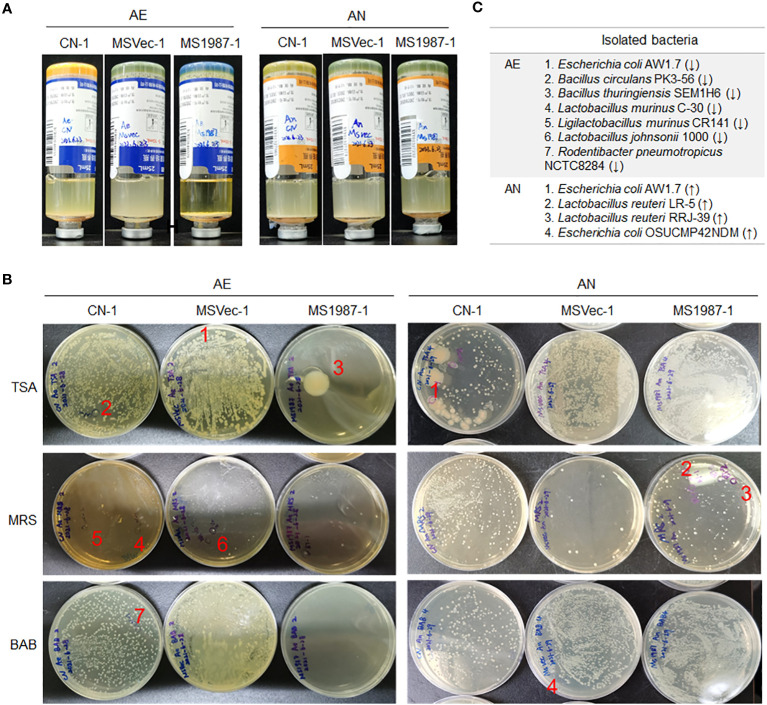
The bacteria in the lung tissues differ between MS1987- and MSVec-infected mice in the cultivation *in vitro*. Equal quantity of the lung tissues (50 mg) was collected from each mouse for MS1987 and MSVec group and homogenized in 1 mL sterile PBS buffer. Forty microliters of the homogenate from each mouse of one group were mixed together. Then half of the mixture was injected into the aerobic blood enrichment culture, and the other half was injected into the anaerobic blood enrichment culture. **(A)** The bacteria cultured and enriched in aerobic and anaerobic conditions. **(B)** The bacteria cultured in various plates. The labeled colonies which were inoculated in the liquid medium and identified by sequencing the V3 region of 16S rRNA. **(C)** The identified bacteria with different abundance between MS1987 and MSVec groups. The arrows ↑ and ↓ represented the bacteria increased and decreased respectively in the MS1987 group compared to the MSVec group. MS1987, *M. smegmatis* overexpressing Rv1987 protein; MSVec, *M. smegmatis* carrying empty pVV2 vector; CN, uninfected control; AE, aerobic culture; AN, anaerobic culture. CN-1, MSVec-1 and MS1987-1 represented the samples collected at day 9 post-infection.

Because cultivation is also an effective method in bacterial isolation and identification which can roughly reflect the composition of aerobic and anaerobic bacteria, the lung microbiota of MS1987- and MSVec-infected mice were cultured both in aerobic and anaerobic conditions for 5 days. The results showed that the aerobic bacteria in the culture bottle of MS1987 group were less than that of the MSVec group while the anaerobic bacteria looked similar (**Figure 2A**), suggesting that the anaerobic bacteria were relatively increased in MS1987-infected mice. The colonies which were different in the numbers between MS1987 and MSVec groups in TSA, BAB and MRS agar plates (**Figure 2B**) were identified by amplifying and sequencing of their V3 regions of 16S rRNA. Seven aerobic bacteria were isolated and identified to be decreased in MS1987-infected mouse lung, including *Escherichia coli* AW1.7, *Bacillus circulans* PK3-56, *Bacillus thuringiensis* SEM1H6, *Lactobacillus murinus* C-30, *Ligilactobacillus murinus* CR141, *Lactobacillus johnsonii* 1000, and *Rodentibacter pneumotropicus* NCTC8284. Four anaerobic bacteria, *Escherichia coli* AW1.7, *Escherichia coli* OSUCMP42NDM, *Lactobacillus reuteri* LR-5 and *Lactobacillus reuteri* RRJ-39, were identified to be increased in MS1987-infected mouse lung (**Figure 2C**).

### Rv1987-attenuated inflammatory response contributes to lung microbiota dysbiosis and facilitates mycobacterial survival at secondary-infection

To clarify whether above microbiota changes in MS1987-infected mouse lung were the direct effects of MS1987 bacteria, three significantly altered bacteria in MS1987-infected mouse lung, *E. coli* (increased, isolated from anaerobic conditions), *D. acidovorans* (increased), and *R. pickettii* (decreased), were selected to perform the following *in vitro* experiments. The growth of *E. coli*, *D. acidovorans* and *R. pickettii in vitro* were tested in the presence of the secretory metabolites of MS1987 or MSVec. The results showed that both metabolites of MS1987 and MSVec promoted the growth of *E. coli*, *D. acidovorans* and diminished the growth of *R. pickettii*, but there was no significant difference between MS1987 and MSVec groups (**Supplementary Figure 2**), indicating that the lung microbiota dysbiosis in MS1987-infected mice was not due to the different secretory metabolites of MS1987 and MSVec bacteria.

**Figure 3:**
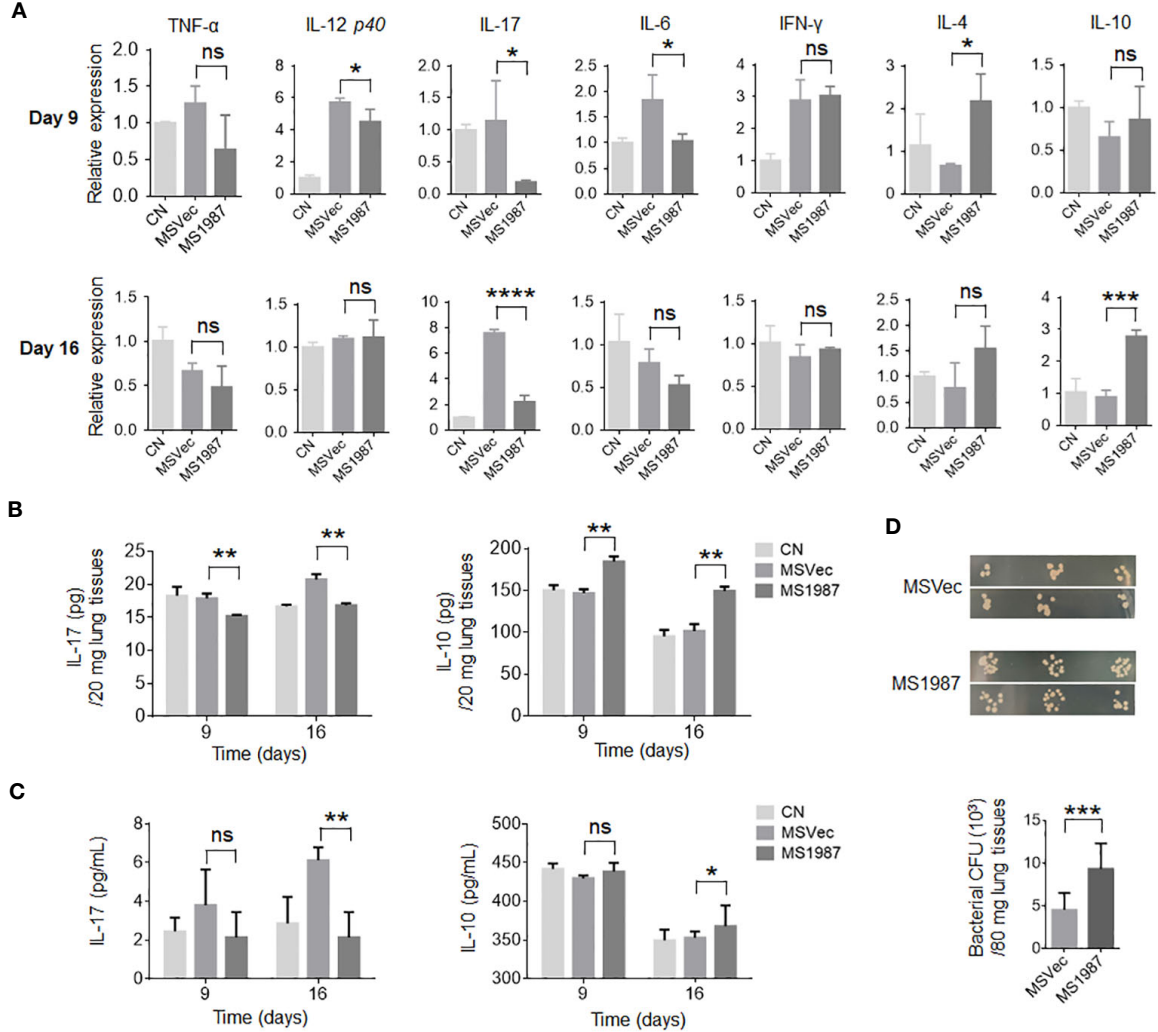
Rv1987 attenuates inflammatory response in mouse lung. **(A)** The relative mRNA expression of pro-inflammatory and anti-inflammatory cytokines in the lung of MS1987- or MSVec-infected mice at day 9 and 16 post-infection. **(B)** The protein level of cytokines IL-17 and IL-10 in the lung of MS1987- or MSVec-infected mice at day 9 and 16 post-infection. **(C)** The cytokine levels in the sera of MS1987- or MSVec-infected mice at day 9 and 16 post-infection. **(D)** The bacterial CFU in mouse lung. The mice were firstly infected with MS1987 or MSVec and then all re-infected by MSVec at day 9 post-primary-infection. The results were from 5 mice of each group and shown as mean ± SD. In figure **(A)** and **(B)**, the difference was compared by one-way ANOVA analysis with *post-hoc* Tukey’s multiple comparisons between MS1987, MSVec and CN groups. In figure **(C)**, the difference was analyzed by unpaired *t*-test between MS1987 and MSVec groups. *p < 0.05; **p < 0.01; ***p < 0.001; ****p < 0.0001; ns, no significant difference. MS1987, *M. smegmatis* overexpressing Rv1987 protein; MSVec, *M. smegmatis* carrying empty vector; CN, uninfected control; CFU, colony forming unit.

Since there is a close association between microbiota and local immune, we then analyzed the immune status of MS1987- and MSVec-infected mouse lung both at mRNA and protein levels. The total RNA was isolated from the lung tissues of MS1987- and MSVec-infected mice and the mRNA level of various cytokines were analyzed by qPCR. It was found that at day 9 post-infection, the inflammatory cytokines IL-12, IL-17 and IL-6 were significantly decreased (*p* < 0.05), while immunosuppressive mediator IL-4 was increased (*p* < 0.05) in MS1987-infected mouse lung compared with MSVec-infected mouse lung (**Figure 3A**). At day 16 post-infection, IL-17 was impaired more obviously (*p* < 0.0001), while immunosuppressive cytokine IL-10 was enhanced significantly (*p* < 0.001). These changes in cytokines indicated an impaired inflammatory response occurred in MS1987-infected mouse lung compared to MSVec-infected mouse lung (**Figure 3A**). The concentration of cytokines IL-17 and IL-10 in lung tissues detected by ELISA (**Figure 3B**) was generally consistent with the results at mRNA level. To clarify whether these changes influenced systemic immune, we detected the expression of IL-17 and IL-10 in mouse sera by ELISA. The results showed that serum IL-17 level was remarkably lower (*p* < 0.01) in MS1987 group compared to MSVec group at day 16 after infection. IL-10 was also increased in the sera of MS1987-infected mice (**Figure 3C**). All these results suggested that Rv1987 protein attenuated the inflammatory response induced by mycobacteria in local lung tissues, which was probably one of the reasons that led to the microbiota dysbiosis in the lung.

To reveal whether the impaired inflammatory response affected mycobacterial survival in the lung, the mice infected by MS1987 or MSVec were both re-infected by MSVec at day 9 post-primary-infection. The results showed that bacterial CFU in the lung of MS1987-infected mice was significantly higher than that in MSVec-infected mice, suggesting the attenuated inflammatory response induced by Rv1987protein facilitated mycobacterial survival at secondary-infection (**Figure 3D**).

### The altered bacteria induce anti-inflammatory responses in mouse lung

**Figure 4:**
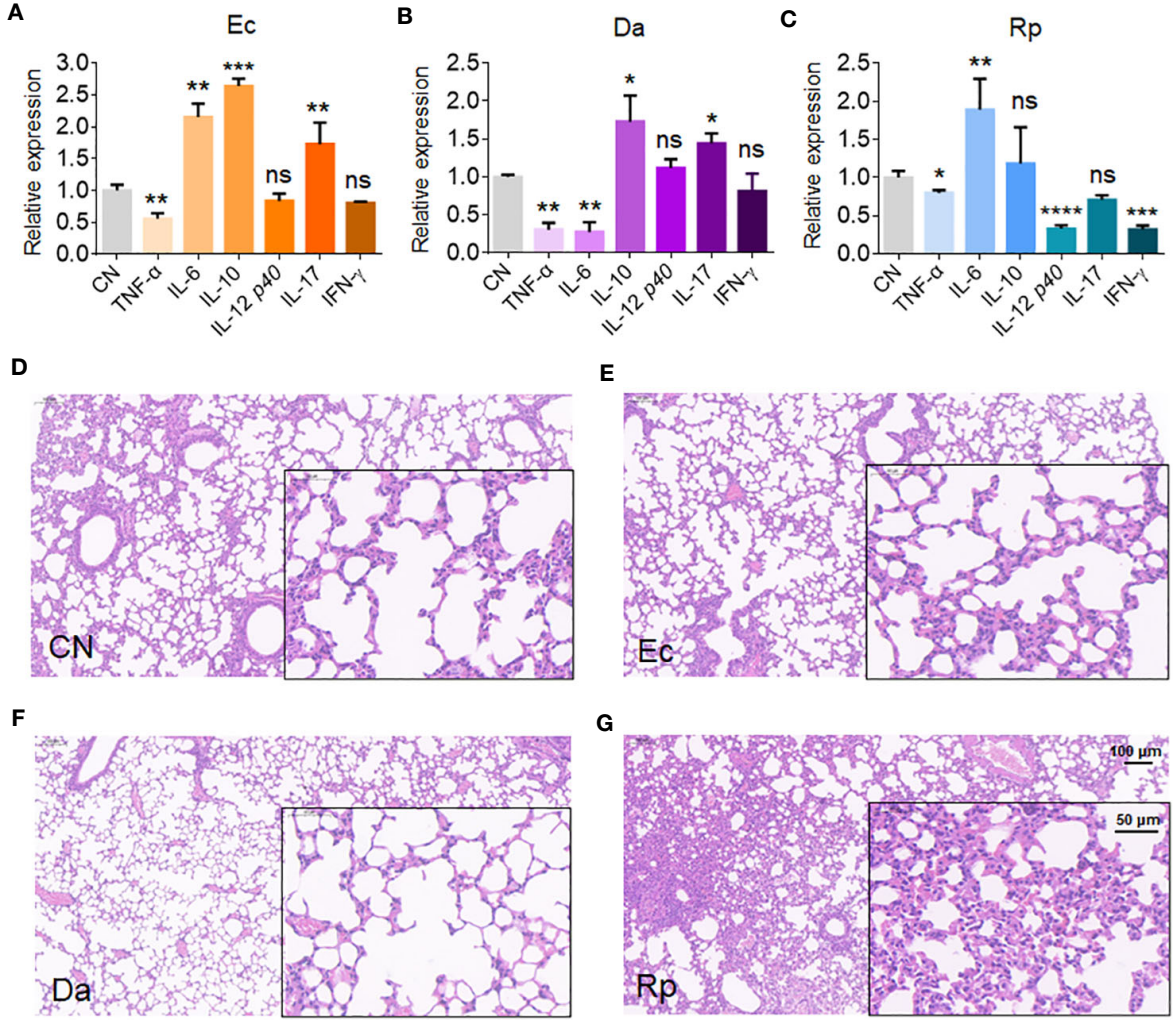
*E. coli*, *D. acidovorans* and *R. pickettii* affect cytokine production and histopathology in mouse lung. The mouse was infected by *E. coli*, *D. acidovorans* or *R. pickettii* at 5 × 10^8^ CFU/day for 4 days and the sera and lung tissues of mice were collected at day 9 post-infection. **(A-C)** The mRNA level of cytokines in the mouse lung infected by *E. coli* **(A)**, *D. acidovorans* **(B)** and *R. pickettii* **(C)**. **(D-G)** The lung histopathology of the mice without infection **(D)**, or infected by *E. coli-* **(E)**, *D. acidovorans-* **(F)** and *R. pickettii* **(G)**. The results were shown as mean ± SD from 5 mice and compared between each infected group with CN group by unpaired *t*-test. *p < 0.05; **p < 0.01; ***p < 0.001; ****p < 0.0001; ns, no significant difference. Ec, *Escherichia coli* AW1.7; Da, *Delftia acidovorans*; Rp, *R. pickettii*; CN, uninfected control.

To understand the effects of Rv1987-induced microbiota dysbiosis on the lung homeostasis of the host, the mice were inhaled independently by three obviously altered bacteria in MS1987-infected mice, *E. coli* (increased, isolated from anaerobic conditions), *D. acidovorans* (increased) and *R. pickettii* (decreased). The infection lasted 4 days and the sera and lung tissues of mice were collected at day 9 post-infection. The results showed that anaerobic *E. coli* led to the production of both inflammatory cytokines IL-6 and IL-17 and anti-inflammatory cytokine IL-10 (**Figure 4A**). *D. acidovorans* inhibited the synthesis of inflammatory cytokines TNF-α and IL-6, meanwhile stimulated the production of anti-inflammatory cytokine IL-10 (**Figure 4B**). *R. pickettii* impaired the production of most cytokines, but elevated the level of inflammatory cytokine IL-6 significantly (**Figure 4C**). The histological staining showed that compared with non-infected mice (**Figure 4D**), no obvious inflammation such as angiectasis, hyperemia and alveolar septum enlargemen in histological staining was observed in *E. coli*- and *D. acidovorans*-infected mouse lung (**Figures 4E, F**), but inflammation occurred in *R. pickettii*-infected mouse lung with hyperemia and neutrophil infiltration (**Figure 4G**). All these results indicated that the increase of *E. coli* and *D. acidovorans* and the decrease of *R. pickettii* resulted in an anti-inflammatory environment in local lung tissues, which contributed to the survival of mycobacteria in host.

### The altered bacteria promote the growth of mycobacterium and its biofilm

**Figure 5:**
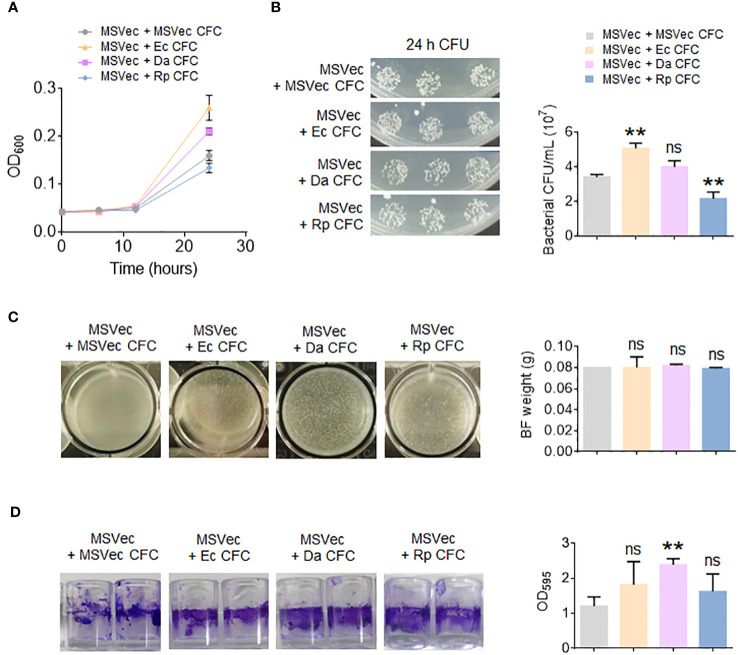
The metabolites of *E. coli*, *D. acidovorans* and *R. pickettii* affect the growth of mycobacterium and its biofilm. MSVec was grown in the glass tubes or the wells in absent or presence of the CFC of *E. coli*, *D. acidovorans* and *R. pickettii*. **(A)** The absorbance at 600 nm (OD_600_) of bacteria grown in glass tubes. **(B)** The CFU counting of bacteria in figure **(A)**. **(C)** The weight of air-liquid layer biofilm grown in the wells. **(D)** The CV staining of the biofilm formed on the side wall of the wells. The results in bar charts were shown as mean ± SD from 3 independent experiments. The differences were compared between the groups in presence or lack of bacterial CFC by unpaired *t*-test. **p < 0.01; ns, no significant difference. MSVec, *M. smegmatis* carrying empty pVV2 vector; Ec, *Escherichia coli* AW1.7; Da, *Delftia acidovorans*; Rp, *R. pickettii*; CFC, cell-free culture; BF, biofilm; CFU, colony forming unit.

To investigate whether the secretory metabolites of altered bacteria have direct effect on mycobacterial growth, the CFC of *E. coli*, *D. acidovorans* and *R. pickettii* were added in the culture of MSVec. The results showed that the metabolites of *E. coli* significantly stimulated mycobacterial growth, while the metabolites of *R. pickettii* inhibited mycobacterial growth (**Figures 5A, B**). Because the biofilm is the key structure for the infection and growth of Mtb in host, the effects of the metabolites of *E. coli*, *D. acidovorans* and *R. pickettii* on the biofilm information of mycobacteria were also tested *in vitro*. The biofilm formed in the air-liquid layer was evaluated by weighing its weight and the biofilm formed on the side wall of the wells was determined by CV staining. The results showed that *E. coli*, *D. acidovorans* and *R. pickettii* had no impacts on the formation of air-liquid layer biofilm between groups (**Figure 5C**), but they all promoted the biofilm growth on the side wall of the wells, especially *D. acidovorans* (*p* < 0.01) (**Figure 5D**).

### The microbiota dysbiosis affects metabolic profiles of mouse lung

**Figure 6:**
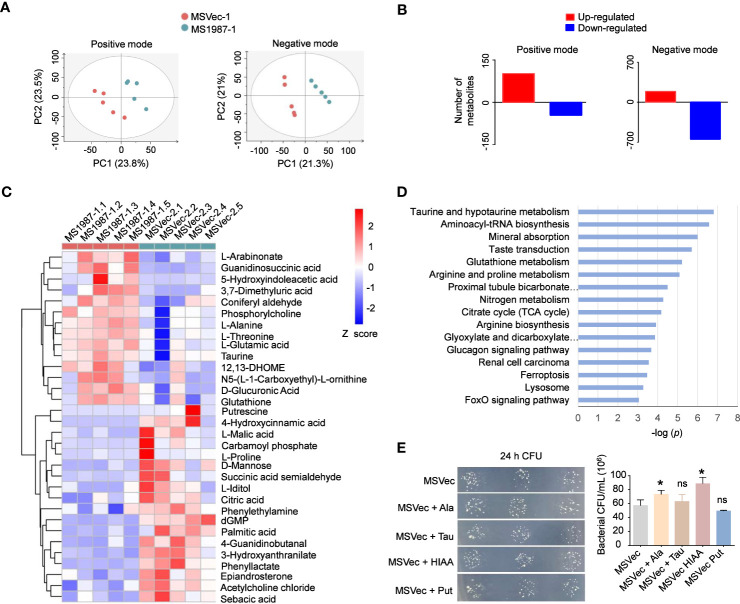
Rv1987-induced immune alterations and microbiota dysbiosis affect metabolic profiles of mouse lung. **(A)** PLS-DA model of the metabolites in the lung of MS1987-infected and MSVec-infected mice. **(B)** The number of altered metabolites in the lung of MS1987-infected mice compared to MSVec-infected mice. **(C)** The significantly altered metabolites in the lung of MS1987-infected mice compared to MSVec-infected mice. The results were from 5 mice for each group and the difference was compared by unpaired *t*-test between MS1987 and MSVec groups. **(D)** KEGG pathway analysis of different metabolites. **(E)** The effects of some metabolites on mycobacterial growth. The results were shown as mean ± SD from 3 independent experiments and compared by unpaired *t*-test between MSVec group and other groups. *p < 0.05; ns, no significant difference. MS1987, *M. smegmatis* overexpressing Rv1987 protein; MSVec, *M. smegmatis* carrying empty vector. Ala, L-Alanine; Tau, Taurine; HIAA, 5-Hydroxyindoleacetic acid; Put, Putrescine.

To clarify whether the dysbiosis of lung microbiota had impacts on the local microenvironments and correspondingly influenced the mycobacterial survival in the host lung, the metabolite profiles of MS1987- and MSVec-infected mouse lung were analyzed by untargeted liquid chromatography (LC)-mass spectrometry (MS)/MS. Partial least squares discriminant analysis (PLS-DA) showed that the plots of MS1987 group were significantly separated from those of MSVec group in both positive mode (R2Y = 0.944, Q2 = 0.542) and negative mode (R2Y = 0.999, Q2 = 0.936) in (**Figure 6A**), indicating that there was a significant difference in metabolites between MS1987- and MSVec-infected mouse lung. In positive mode, it was found that 102 metabolites were up-regulated and 46 metabolites were down-regulated in MS1987-infected mouse lung compared to MSVec-infected mouse lung. In negative mode, more alterations were revealed with 188 up-regulated metabolites and 647 down-regulated metabolites (**Figure 6B**). Among them, 32 metabolites were finally confirmed by further validation of MWs errors and *p* values (**Figure 6C**). Classification of these metabolites found that 16 pathways were significantly different between MS1987 and MSVec groups (**Figure 6D**). The increased amino acid metabolism and aminoacyl-tRNA biosynthesis implied an enhanced remodeling in MS1987-infeced mice compared to MSVec-infected mice. The fluency of tricarboxylic acid (TCA) cycle without accumulation of citric acid indicated the energy were relatively rich in MS1987-infected mouse lung. All these results suggested that the dysbiosis of lung microbiota induced by Rv1987 protein constructed a “relaxed” and “friendly” metabolic environment for mycobacterial survival and replication. To prove that, we selected four significantly altered metabolites in MS1987-infected mice, L-alanine, taurine, 5-hydroxyindoleacetic acid and putrescine, to perform *in vitro* experiments to study their effects on mycobacterial growth. Among these four metabolites, 5-hydroxyindoleacetic acid was increased mostly in MS1987-infected mice compared to MSVec-infected mice, while putrescine was decreased mostly. L-alanine had the smallest *p* value among all metabolites. Taurine was associated with the pathway of “Taurine and hypotaurine metabolism” which was significantly altered in MS1987-infected mice with the smallest *p* value (**Figure 6D**). The results showed that two metabolites which were upregulated in MS1987-infected mouse lung, L-alanine and 5-hydroxyindoleacetic acid, had the abilities to promote the growth of mycobacteria (**Figure 6E**). All these results indicated that the immune alterations and microbiota dysbiosis induced by Rv1987 protein affected the metabolic microenvironments in host, which may be another factor promoting mycobacterial survival in host lung.

## Discussion

In this study, *M. smegmatis* airway infection model was used to study the effect of Mtb Rv1987 protein on the immune response and microbiota composition of host lung. As a non-pathogenic and fast-growing mycobacterium, *M. smegmatis* has similar cellular structure to Mtb and is always used as a model strain to study the function of Mtb proteins (T et al., 2020). Because there is no ortholog of Rv1987, *M. smegmatis* is an ideal model for investigation of Rv1987 functions. In order to increase the level of Rv1987 protein in local lung tissues, a high dose of *M. smegmatis* was used to infect mice as we reported previously (Sha et al., 2017). It might have a different impact from *M. tuberculosis* infection at an appropriate dose, however, the effect of Rv1987 on host immune and microbiota composition can be concluded by comparing the results from Rv1987 overexpressing *M. smegmatis* strain with the results from the control *M. smegmatis* strain.

Mtb Rv1987 protein was revealed to affect the immune state and microbiota composition of host lung. The cytokine levels were significantly altered in the lung tissues of MS1987-infected mice compared to the control group. As we known, the cytokines produced by alveolar epithelial cells and immune cells reflect the immune state of lung and are key factors in the host defense of pathogens. During bacterial and viral infections, local sensor cells in respiratory tract, including airway epithelia cells, alveolar macrophages and dendritic cells, firstly response to pathogens and secret the first-order cytokines, such as type I and III IFN, IL-6, TNF-α, IL-12, IL-25, IL-33, IL-1β, etc. (Happel et al., 2005; Liu, 2005; Khaitov et al., 2009; Barlow et al., 2013). These cytokines directly drive effector cells such as cytotoxic T cells and neutrophils to clear the pathogens, or trigger the tissue-resistant lymphocytes to produce second-order cytokines. Among these lymphocytes, NK, NKT, Th1, γδT, ILC3 and Th17 cells response to IL-12 and IL-1β, lead to the secretion of inflammatory cytokines IFN-γ, IL-17 and IL-22 which enhance the microbiocidal activation of effector cells (Iwasaki et al., 2017). On the other hand, in response to IL-25 and IL-33, ILC3 and Th2 cells produce anti-inflammatory cytokines, such as IL-4, IL-10 and IL-13, which impair the pathogen elimination by effector cells (Iwasaki et al., 2017). In this study, we found that compared to MSVec-infected mouse, MS1987-infected mouse lung exhibited an impaired inflammatory response with decreased TNF-α, IL-12, and IL-17 and increased IL-4 and IL-10. These changes in local tissues may be one of the reasons that Rv1987 protein enhances the mycobacterial survival in mouse lung (Sha et al., 2017), and also the reason of microbiota alterations in this study.

The alterations of host immune state may result in microbiota dysbiosis. It was reported that HIV patients receiving antiretroviral therapy had an enrichment of oral anaerobes in the lung and remained highly susceptible to tuberculosis (Segal et al., 2017). The anaerobic bacterial fermentation products, butyrate and other short-chain fatty acids (SCFAs), inhibited IFN-γ and IL-17 production (Segal et al., 2017), promoted IL-10 secretion (Sun et al., 2018), and induced regulatory T cells differentiation (Furusawa et al., 2013; Smith et al., 2013). Obviously, these changes were adverse for host to eliminate Mtb. In this study, Rv1987 protein attenuated the local pro-inflammatory responses, which inevitably led to the microbiota dysbiosis in the lung. The diversity of bacteria was decreased and anaerobic bacteria were relatively increased in MS1987-infected mouse lung. It was noted that besides immune responses, the disruption of epithelial barrier (Brune et al., 2015) and the functional changes of alveolar epithelial cells (Ruaro et al., 2021), may also influence the stability of lung microbiota. It is worthy to study further whether Rv1987 protein has impacts on physical barrier and epithelial cells through specific receptors and signaling.

Considering that immune state and lung microbiota play important roles in maintaining lung homeostasis, the metabolome of the lung tissues of MS1987- and MSVec-infected mice were analyzed by LC-MS/MS. The results showed that the ammino acid metabolism and energy metabolism were enhanced in MS1987-infected mouse lung, indicating a kindly microenvironment for mycobacterial survival was generated in the host lung. The enhanced glutathione metabolism with an increase of glutathione in MS1987-infected mouse lung may be the results of increased mycobacterial infection. Generally, glutathione is believed to have protective ability against microbial infection through direct antimycobacterial effects and enhancing the function of immune cells (Morris et al., 2013). It can also protect host cells against the toxic effects of reactive oxygen species and reactive nitrogen species when infection occurs (Seres et al., 2000). In some TB patients, the level of glutathione is increased (Petrillo et al., 2022). Further studies on the altered metabolites *in vitro* found that some of them can influence mycobacterial growth directly (**Figure 6E**). L-Alanine, which was increased in MS1987-infected mouse lung, significantly stimulated mycobacterial growth *in vitro*. It was probably because alanine can be taken as nitrogen source of Mtb (Agapova et al., 2019). In contrast, putrescine, which was decreased in MS1987-infected mouse lung, inhibited mycobacterial growth slightly. The reason may be that putrescine can decrease the permeability of mycobacteria and shift mycobacteria to non-replication state (Sarathy et al., 2013). It is tempting to speculate that the host metabolite alterations induced by microbiota dysbiosis also contributed to mycobacterial evasion, although these results were from *in vitro* experiments and need further investigations in future studies.

To further understand the effects of the microbiota dysbiosis on the lung immune state of host, we focused our attention on the top ten bacteria in abundance in MS1987-infected mice. Six bacteria, including *R. pickettii*, *C. aquatica*, *P. azotoformans*, *E. coli*, *D. acidovorans*, *Parabacteroides* sp. CT06, were shown to be significantly altered in MS1987-infeced mouse lung at day 9 post-infection (**Figure 1E**). Among them, aerobic bacteria *D. acidovorans* and *R. pickettii* and anaerobic bacterium *E. coli* were selected to perform the following studies independently because they were significantly changed in MS1987-infected mice. In addition, they were all reported to exist in the lung tissues. Usually, *E. coli* is not considered as major pathogens for lung infectious diseases (Croxen and Finlay, 2010). It always infects urinary tract, bloodstream, cerebral spinal fluid, and peritoneum. However, in recent years, *E. coli* is found to be closely related to hospital acquired infections, especially the ventilator-assisted pneumonia (Fihman et al., 2015; La Combe et al., 2019), which indicates that *E. coli* exists in healthy lung tissues and can cause diseases in some conditions. *D. acidovorans* is a nonpathogenic environmental organism and usually believed to have not clinical significance. However, it is revealed to cause diseases in immunocompetent and immunocompromised patients recently, including septic pulmonary embolism (Patel et al., 2019) and B cell lymphoblastic leukemia patients (Bilgin et al., 2015). *R. pickettii* is another bacterium that is reported to present in lung tissues and be associated with lung diseases, such as lobar pneumonia (Pan et al., 2011) and mesotheliomas (Higuchi et al., 2021). In this study, anaerobic *E. coli* and aerobic *D*. *acidovorans* were found to be increased in MS1987-infected mouse lung. Infection of mice by *E. coli* and *D*. *acidovorans* alone revealed that they played regulatory roles in the host lung by stimulating the production of anti-inflammatory cytokines. In contrast, *R. pickettii* caused an acute inflammation in the lung, therefore its decrease in MS1987-infected mouse lung was also helpful for constructing an anti-inflammatory microenvironment. Obviously, the impaired inflammatory responses facilitated the mycobacterial survival in local tissues. All these results suggested that the dysbiosis of lung microbiota induced by Rv1987 protein further disrupted the lung homeostasis, which provided a “hotbed” for mycobacterial growth.

## Conclusions

The bidirectional interactions between mycobacterium and microbiota in host lung were observed in this study. Rv1987 protein as an Mtb virulence factor attenuated inflammatory response in mouse lung and led to the dysbiosis of lung microbiota consequently. As an accomplice, the altered microbiota enhanced the disruption of lung homeostasis and stimulated the mycobacterial growth directly, which contributed to the mycobacterial survival in host lung.

## Data availability statement

The data presented in the study are deposited in the GenBank repository, accession number PRJNA1020094 (https://www.ncbi.nlm.nih.gov/bioproject/1020094), and the MetaboLights repository, accession number MTBLS8657 (https://www.ebi.ac.uk/metabolights/MTBLS8657).

## Ethics statement

All animal experiments conformed to the NIH Guide for Care and Use of Laboratory Animals and were approved by the Committee on the Ethics of Animal Experiments of Dalian Medical University. Permission number: SYXK (Liao) 2018-0007.

## Author contributions

YL: Formal Analysis, Investigation, Methodology, Writing – review & editing. JZ: Formal Analysis, Investigation, Methodology, Writing – review & editing. GL: Data curation, Software, Writing – review & editing. JH: Validation, Writing – review & editing, Software. WW: Data curation, Software, Writing – review & editing. GD: Data curation, Validation, Writing – review & editing. YM: Conceptualization, Writing – review & editing. SS: Conceptualization, Funding acquisition, Investigation, Project administration, Writing – original draft.
