# Supplementary material for: Mycobacterium tuberculosis Rv1987 protein attenuates inflammatory response and consequently alters microbiota in mouse lung
Source: Front Cell Infect Microbiol. 2023 Nov 1;13:1256866. doi: 10.3389/fcimb.2023.1256866 (PMC10646435; doi:10.3389/fcimb.2023.1256866)
Supplement: Supplementary file 2 [file DataSheet_2.docx]

Source data for Figure 2

The sequencing results of the V3 region of 16S rRNA for bacterial colonies.

**AE-1**

GCTTGCTGCTTCGCTGACGAGTGGCGGACGGGTGAGTAATGTCTGGGAAA

CTGCCTGATGGAGGGGGATAACTACTGGAAACGGTAGCTAATACCGCATA

ACGTCGCAAGACCAAAGAGGGGGACCTTCGGGCCTCTTGCCATCGGATGT

GCCCAGATGGGATTAGCTAGTAGGTGGGGTAACGGCTCACCTAGGCGACG

ATCCCTAGCTGGTCTGAGAGGATGACCAGCCACACTGGAACTGAGACACG

GTCCAGACTCCTACGGGAGGCAGCAGTGGGGAATATTGCACAATGGGCGC

AAGCCTGATGCAGCCATGCCGCGTGTATGAAGAAGGCCTTCGGGTTGTAA

AGTACTTTCAGCGGGGAGGAAGGGAGTAAAGTTAATACCTTTGCTCATTG

ACGTTACCCGCAGAAGAAGCACCGGCTAACTCCGTGCCAGCAGCCGCGGT

AATACGGAGGGTGCAAGCGTTAATCGGAATTACTGGGCGTAAAGCGCACG

CAGGCGGTTTGTTAAGTCAGATGTGAAATCCCCGGGCTCAACCTGGGAAC

TGCATCTGATACTGGCAAGCTTGAGTCTCGTAGAGGGGGGTAGAATTCCA

GGTGTAGCGGTGAAATGCGTAGAGATCTGGAGGAATACCGGTGGCGAAGG

CGGCCCCCTGGACGAAGACTGACGCTCAGGTGCGAAAGCGTGGGGAGCAA

ACAGGATTAGATACCCTGGTAGTCCACGCCGTAAACGATGTCGACTTGGA

GGTTGTGCCCTTGAGGCGTGGCTTCCGGAGCTAACGCGTTAAGTCGACCG

CCTGGGGAGTACGGCCGCAAGGTTAAAACTCAAATGAATTGACGGGGGCC

CGCACAAGCGGTGGAGCATGTGGTTTAATTCGATGCAACGCGAAGAACCT

TACCTGGTCTTGACATCCACGGAAGTTTTCAGAGATGAGAATGTGCCTTC

GGGAACCGTGAGACAGGTGCTGCATGGCTGTCGTCAGCTCGTGTTGTGAA

ATGTTGGGTTAAGTCCCGCAACGAGCGCAACCCTTATCCTTTGTTGCCAG

CGGTCCGGCCGGGAACTCAAAGGAGACTGCCAGTGATAAACTGGAGGAAG

GTGGGGATGACGTCAAGTCATCATGGCCCTTACGACCAGGGCTACACACG

TGCTACAATGGCGCATACAAAGAGAAGCGACCTCGCGAGAGCAAGCGGAC

CTCATAAAGTGCGTCGTAGTCCGGATTGGAGTCTGCAACTCGACTCCATG

AAGTCGGAATCGCTAGTAATCGTGGATCAGAATGCCACGGTGAATACGTT

CCCGGGCCTTGTACACACCGCCCGTCACACCATGGGAGTGGGTTGCAAAA

GAAGTAGGTAGCTTAACCTTCGGGAGGGCG

**AE-2**

GCAGTCGAGCGGACTTTAAAAGCTTGCTTTTAAAGTTAGCGGCGGACGGG

TGAGTATCACGTGGGCAACCTGCCTGTAAGACTGGGATAACTTCGGGAAA

CCGGAGCTAATACCGGATAATCCTTTTCCTCTCATGAGGAAAAGCTGAAA

GACGGTTTACGCTGTCACTTACAGATGGGCCCGCGGCGCATTAGCTAGTT

GGTGAGGTAACGGCTCACCAAGGCGACGATGCGTAGCCGACCTGAGAGGG

TGATCGGCCACACTGGGACTGAGACACGGCCCAGACTCCTACGGGAGGCA

GCAGTAGGGAATCTTCCGCAATGGACGAAAGTCTGACGGAGCAACGCCGC

GTGAGTGATGAAGGTTTTCGGATCGTAAAACTCTGTTGTTAGGGAAGAAC

AAGTACAAGAGTAACTGCTTGTACCTTGACGGTACCTAACCAGAAAGCCA

CGGCTAACTACGTGCCAGCAGCCGCGGTAATACGTAGGTGGCAAGCGTTG

TCCGGAATTATTGGGCGTAAAGCGCGCGCAGGCGGTCCTTTAAGTCTGAT

GTGAAAGCCCACGGCTCAACCGTGGAGGGTCATTGGAAACTGGGGGACTT

GAGTGCAGAAGAGAAGAGTGGAATTCCACGTGTAGCGGTGAAATGCGTAG

AGATGTGGAGGAACACCAGTGGCGAAGGCGACTCTTTGGTCTGTAACTGA

CGCTGAGGCGCGAAAGCGTGGGGAGCAAACAGGATTAGATACCCTGGTAG

TCCACGCCGTAAACGATGAGTGCTAAGTGTTAGAGGGTTTCCGCCCTTTA

GTGCTGCAGCAAACGCATTAAGCACTCCGCCTGGGGAGTACGGCCGCAAG

GCTGAAACTCAAAGGAATTGACGGGGGCCCGCACAAGCGGTGGAGCATGT

GGTTTAATTCGAAGCAACGCGAAGAACCTTACCAGGTCTTGACATCCTCT

GACACTCCTAGAGATAGGACGTTCCCCTTCGGGGGACAGAGTGACAGGTG

GTGCATGGTTGTCGTCAGCTCGTGTCGTGAGATGTTGGGTTAAGTCCCGC

AACGAGCGCAACCCTTGATCTTAGTTGCCAGCATTCAGTTGGGCACTCTA

AGGTGACTGCCGGTGACAAACCGGAGGAAGGTGGGGATGACGTCAAATCA

TCATGCCCCTTATGACCTGGGCTACACACGTGCTACAATGGATGGTACAA

AGGGCAGCAAAACCGCGAGGTCGAGCAAATCCCATAAAACCATTCTCAGT

TCGGATTGTAGGCTGCAACTCGCCTACATGAAGCTGGAATCGCTAGTAAT

CGCGGATCAGCATGCCGCGGTGAATACGTTCCCGGGCCTTGTACACACCG

CCCGTCACACCACGAGAGTTTGTAACACCCGAAGTCGGTGGGGTAACCTT

TGGAGCCAGCCGCCTAA

**AE-3**

AAGTCGAGCGAATGGATTAAGAGCTTGCTCTTATGAAGTTAGCGGCGGAC

GGGTGAGTAACACGTGGGTAACCTGCCCATAAGACTGGGATAACTCCGGG

AAACCGGGGCTAATACCGGATAACATTTTGAACTGCATGGTTCGAAATTG

AAAGGCGGCTTCGGCTGTCACTTATGGATGGACCCGCGTCGCATTAGCTA

GTTGGTGAGGTAACGGCTCACCAAGGCAACGATGCGTAGCCGACCTGAGA

GGGTGATCGGCCACACTGGGACTGAGACACGGCCCAGACTCCTACGGGAG

GCAGCAGTAGGGAATCTTCCGCAATGGACGAAAGTCTGACGGAGCAACGC

CGCGTGAGTGATGAAGGCTTTCGGGTCGTAAAACTCTGTTGTTAGGGAAG

AACAAGTGCTAGTTGAATAAGCTGGCACCTTGACGGTACCTAACCAGAAA

GCCACGGCTAACTACGTGCCAGCAGCCGCGGTAATACGTAGGTGGCAAGC

GTTATCCGGAATTATTGGGCGTAAAGCGCGCGCAGGTGGTTTCTTAAGTC

TGATGTGAAAGCCCACGGCTCAACCGTGGAGGGTCATTGGAAACTGGGAG

ACTTGAGTGCAGAAGAGGAAAGTGGAATTCCATGTGTAGCGGTGAAATGC

GTAGAGATATGGAGGAACACCAGTGGCGAAGGCGACTTTCTGGTCTGTAA

CTGACACTGAGGCGCGAAAGCGTGGGGAGCAAACAGGATTAGATACCCTG

GTAGTCCACGCCGTAAACGATGAGTGCTAAGTGTTAGAGGGTTTCCGCCC

TTTAGTGCTGAAGTTAACGCATTAAGCACTCCGCCTGGGGAGTACGGCCG

CAAGGCTGAAACTCAAAGGAATTGACGGGGGCCCGCACAAGCGGTGGAGC

ATGTGGTTTAATTCGAAGCAACGCGAAGAACCTTACCAGGTCTTGACATC

CTCTGAAAACCCTAGAGATAGGGCTTCTCCTTCGGGAGCAGAGTGACAGG

TGGTGCATGGTTGTCGTCAGCTCGTGTCGTGAGATGTTGGGTTAAGTCCC

GCAACGAGCGCAACCCTTGATCTTAGTTGCCATCATTAAGTTGGGCACTC

TAAGGTGACTGCCGGTGACAAACCGGAGGAAGGTGGGGATGACGTCAAAT

CATCATGCCCCTTATGACCTGGGCTACACACGTGCTACAATGGACGGTAC

AAAGAGCTGCAAGACCGCGAGGTGGAGCTAATCTCATAAAACCGTTCTCA

GTTCGGATTGTAGGCTGCAACTCGCCTACATGAAGCTGGAATCGCTAGTA

ATCGCGGATCAGCATGCCGCGGTGAATACGTTCCCGGGCCTTGTACACAC

CGCCCGAAACGCCACGAGAGTTTGTAACACCCGAAGTCGGTGGGGTAACC

TTTTTGGAGCCAGCC

**AE-4**

TGGCGAACGGGTGAGTAACACGTGGGCAACCTGCCCAAAAGAGGGGGATA

ACACTTGGAAACAGGTGCTAATACCGCATAACCATAGTTACCGCATGGTA

ACTATGTAAAAGGTGGCTATGCTACCGCTTTTGGATGGGCCCGCGGCGCA

TTAGCTAGTTGGTGGGGTAAAGGCTTACCAAGGCAATGATGCGTAGCCGA

ACTGAGAGGTTGATCGGCCACATTGGGACTGAGACACGGCCCAAACTCCT

ACGGGAGGCAGCAGTAGGGAATCTTCCACAATGGGCGAAAGCCTGATGGA

GCAACGCCGCGTGGGTGAAGAAGGTCTTCGGATCGTAAAACCCTGTTGTT

AGAGAAGAAAGTGCGTGAGAGTAACTGTTCACGTTTCGACGGTATCTAAC

CAGAAAGCCACGGCTAACTACGTGCCAGCAGCCGCGGTAATACGTAGGTG

GCAAGCGTTATCCGGATTTATTGGGCGTAAAGGGAACGCAGGCGGTCTTT

TAAGTCTGATGTGAAAGCCTTCGGCTTAACCGGAGTAGTGCATTGGAAAC

TGGGAGACTTGAGTGCAGAAGAGGAGAGTGGAACTCCATGTGTAGCGGTG

AAATGCGTAGATATATGGAAGAACACCAGTGGCGAAAGCGGCTCTCTGGT

CTGTAACTGACGCTGAGGTTCGAAAGCGTGGGTAGCAAACAGGATTAGAT

ACCCTGGTAGTCCACGCCGTAAACGATGAATGCTAAGTGTTGGAGGGTTT

CCGCCCTTCAGTGCTGCAGCTAACGCAATAAGCATTCCGCCTGGGGAGTA

CGACCGCAAGGTTGAAACTCAAAGGAATTGACGGGGGCCCGCACAAGCGG

TGGAGCATGTGGTTTAATTCGAAGCAACGCGAAGAACCTTACCAGGTCTT

GACATCTTTTGACAATCCTAGAGATAGGACTTTCCCTTCGGGGACAAAAT

GACAGGTGGTGCATGGTTGTCGTCAGCTCGTGTCGTGAGATGTTGGGTTA

AGTCCCGCAACGAGCGCAACCCTTATTGTTAGTTGCCAGCATTAAGTTGG

GCACTCTAGCAAGACTGCCGGTGACAAACCGGAGGAAGGTGGGGATGACG

TCAAATCATCATGCCCCTTATGACCTGGGCTACACACGTGCTACAATGGA

CGGTACAACGAGTCGCAAGACCGCGAGGTTTAGCAAATCTCTTAAAGCCG

TTCTCAGTTCGGATTGTAGGCTGCAACTCGCCTACATGAAGTCGGAATCG

CTAGTAATCGCGGATCAGCATGCCGCGGTGAATACGTTCCCGGGCCTTGT

ACACACCGCCCGACACACCATGAGAGTTTGTAACACCCAAAGCCGGTGGG

GTAACCTTTGGAGCCAGC

**AE-5**

CAAGTCGAACGAAACTTCTTTATCACCGAGTGCTTGCACTCACCGATAAA

GAGTTGAGTGGCGAACGGGTGAGTAACACGTGGGCAACCTGCCCAAAAGA

GGGGGATAACACTTGGAAACAGGTGCTAATACCGCATAACCATAGTTACC

GCATGGTAACTATGTAAAAGGTGGCTATGCTACCGCTTTTGGATGGGCCC

GCGGCGCATTAGCTAGTTGGTGGGGTAAAGGCTTACCAAGGCAATGATGC

GTAGCCGAACTGAGAGGTTGATCGGCCACATTGGGACTGAGACACGGCCC

AAACTCCTACGGGAGGCAGCAGTAGGGAATCTTCCACAATGGGCGAAAGC

CTGATGGAGCAACGCCGCGTGGGTGAAGAAGGTCTTCGGATCGTAAAACC

CTGTTGTTAGAGAAGAAAGTGCGTGAGAGTAACTGTTCACGTTTCGACGG

TATCTAACCAGAAAGCCACGGCTAACTACGTGCCAGCAGCCGCGGTAATA

CGTAGGTGGCAAGCGTTATCCGGATTTATTGGGCGTAAAGGGAACGCAGG

CGGTCTTTTAAGTCTGATGTGAAAGCCTTCGGCTTAACCGGAGTAGTGCA

TTGGAAACTGGGAGACTTGAGTGCAGAAGAGGAGAGTGGAACTCCATGTG

TAGCGGTGAAATGCGTAGATATATGGAAGAACACCAGTGGCGAAAGCGGC

TCTCTGGTCTGTAACTGACGCTGAGGTTCGAAAGCGTGGGTAGCAAACAG

GATTAGATACCCTGGTAGTCCACGCCGTAAACGATGAATGCTAAGTGTTG

GAGGGTTTCCGCCCTTCAGTGCTGCAGCTAACGCAATAAGCATTCCGCCT

GGGGAGTACGACCGCAAGGTTGAAACTCAAAGGAATTGACGGGGGCCCGC

ACAAGCGGTGGAGCATGTGGTTTAATTCGAAGCAACGCGAAGAACCTTAC

CAGGTCTTGACATCTTTTGCCAATCCTAGAGATAGGACTTTCCCTTCGGG

GACAAAATGACAGGTGGTGCATGGTTGTCGTCAGCTCGTGTCGTGAGATG

TTGGGTTAAGTCCCGCAACGAGCGCAACCCTTATTGTTAGTTGCCAGCAT

TAAGTTGGGCACTCTAGCAAGACTGCCGGTGACAAACCGGAGGAAGGTGG

GGATGACGTCAAATCATCATGCCCCTTATGATCTGGGCTACACACGTGCT

ACAATGGACGGTACAACGAGTCGCAAGACCGCGAGGTTTAGCAAATCTCT

TAAAGCCGTTCTCAGTTCGGATTGTAGGCTGCAACTCGCCTACATGAAGT

CGGAATCGCTAGTAATCGCGGATCAGCATGCCGCGGTGAATACGTTCCCG

GGCCTTGTACACACCGC

**AE-6**

GCAGTCGAGCGAGCTTGCCTAGATGATTTTAGTGCTTGCACTAAATGAAA

CTAGATACAAGCGAGCGGCGGACGGGTGAGTAACACGTGGGTAACCTGCC

CAAGAGACTGGGATAACACCTGGAAACAGATGCTAATACCGGATAACAAC

ACTAGACGCATGTCTAGAGTTTGAAAGATGGTTCTGCTATCACTCTTGGA

TGGACCTGCGGTGCATTAGCTAGTTGGTAAGGTAACGGCTTACCAAGGCA

ATGATGCATAGCCGAGTTGAGAGACTGATCGGCCACATTGGGACTGAGAC

ACGGCCCAAACTCCTACGGGAGGCAGCAGTAGGGAATCTTCCACAATGGA

CGAAAGTCTGATGGAGCAACGCCGCGTGAGTGAAGAAGGGTTTCGGCTCG

TAAAGCTCTGTTGGTAGTGAAGAAAGATAGAGGTAGTAACTGGCCTTTAT

TTGACGGTAATTACTTAGAAAGTCACGGCTAACTACGTGCCAGCAGCCGC

GGTAATACGTAGGTGGCAAGCGTTGTCCGGATTTATTGGGCGTAAAGCGA

GTGCAGGCGGTTCAATAAGTCTGATGTGAAAGCCTTCGGCTCAACCGGAG

AATTGCATCAGAAACTGTTGAACTTGAGTGCAGAAGAGGAGAGTGGAACT

CCATGTGTAGCGGTGGAATGCGTAGATATATGGAAGAACACCAGTGGCGA

AGGCGGCTCTCTGGTCTGCAACTGACGCTGAGGCTCGAAAGCATGGGTAG

CGAACAGGATTAGATACCCTGGTAGTCCATGCCGTAAACGATGAGTGCTA

AGTGTTGGGAGGTTTCCGCCTCTCAGTGCTGCAGCTAACGCATTAAGCAC

TCCGCCTGGGGAGTACGACCGCAAGGTTGAAACTCAAAGGAATTGACGGG

GGCCCGCACAAGCGGTGGAGCATGTGGTTTAATTCGAAGCAACGCGAAGA

ACCTTACCAGGTCTTGACATCCAGTGCAAACCTAAGAGATTAGGTGTTCC

CTTCGGGGACGCTGAGACAGGTGGTGCATGGCTGTCGTCAGCTCGTGTCG

TGAGATGTTGGGTTAAGTCCCGCAACGAGCGCAACCCTTGTCATTAGTTG

CCATCATTAAGTTGGGCACTCTAATGAGACTGCCGGTGACAAACCGGAGG

AAGGTGGGGATGACGTCAAGTCATCATGCCCCTTATGACCTGGGCTACAC

ACGTGCTACAATGGACGGTACAACGAGAAGCGAACCTGCGAAGGCAAGCG

GATCTCTTAAAGCCGTTCTCAGTTCGGACTGTAGGCTGCAACTCGCCTAC

ACGAAGCTGGAATCGCTAGTAATCGCGGATCAGCACGCCGCGGTGAATAC

GTTCCCGGGCCTTGTACACACCGCCCG

**AE-7**

TCGAACGGTAACAGGAGAAAGCTTGCTTTCTTGCTGACGAGTGGCGGACG

GTTGAGTAATGCTTGGGAATCTGGCTTATGGAGGGGGATAACTACTGGAA

ACGGTAGCTAATACCGCATAAGGTCTAAGGACAAAAGGGGGCGTAAGCTC

TTGCCATAAGATGAGCCCAAGTGGGATTAGGTAGTTGGTGAGGTAATGGC

TCACCAAGCCTGCGATCTCTAGCTGGTCTGAGAGGATGACCAGCCACACC

GGGACTGAGACACGGCCCGGACTCCTACGGGAGGCAGCAGTGGGGAATAT

TGCGCAATGGGGGGAACCCTGACGCAGCCATGCCGCGTGAATGAAGAAGG

CCTTCGGGTTGTAAAGTTCTTTCGGTGATGAGGAAGGTGATAAGGTTAAT

ACCCTTATTAATTGACGTTAGTCACAGAAGAAGCACCGGCTAACTCCGTG

CCAGCAGCCGCGGTAATACGGAGGGTGCGAGCGTTAATCGGAATAACTGG

GCGTAAAGGGCACGCAGGCGGATTTTTAAGTGAGGTGTGAAAGCCCCGGG

CTTAACCTGGGAATTGCATTTCAGACTGGGAATCTAGAGTACTTTAGGGA

GGGGTAGAATTCCACGTGTAGCGGTGAAATGCGTAGAGATGTGGAGGAAT

ACCGAAGGCGAAGGCAGCCCCTTGGGAATGTACTGACGCTCATGTGCGAA

AGCGTGGGGAGCAAACAGGATTAGATACCCTGGTAGTCCACGCTGTAAAC

GCTGTCGATTTGGGGGTTGGGCTTTAAGCTTGGCGCCCGTAGCTAACGTG

ATAAATCGACCGCCTGGGGAGTACGGCCGCAAGGTTAAAACTCAAATGAA

TTGACGGGGGCCCGCACAAGCGGTGGAGCATGTGGTTTAATTCGATGCAA

CGCGAAGAACCTTACCTACTCTTGACATCCTAAGAATCCGATAGAGATAT

TGGAGTGCCTTCGGGAACTTAGAGACAGGTGCTGCATGGCTGTCGTCAGC

TCGTGTTGTGAAATGTTGGGTTAAGTCCCGCAACGAGCGCAACCCTTATC

CTTTGTTGCCAGCGATACGGTCGGGAACTCAAAGGAGACTGCCGGTGATA

AACCGGAGGAAGGTGGGGATGACGTCAAGTCATCATGGCCCTTACGAGTA

GGGCTACACACGTGCTACAATGGCGTATACAGAGGGAAGCGAAGCAGCGA

TGTGGAGCGAATCTCATAAAGTGCGTCTAAGTCCGGATTGGAGTCTGCAA

CTCGACTCCATGAAGTCGGAATCGCTAGTAATCGCGAATCAGAATGTCGC

GGTGAATACGTTCCCGGGCCTTGTACACACCGCCCGACAGGCCATGGGAG

TGGGTTGTACCAGAAGTGGATAGCTTAACCGAAAGGGGGGCGTC

**AN-1**

GCTTGCTGCTTCGCTGACGAGTGGCGGACGGGTGAGTAATGTCTGGGAAA

CTGCCTGATGGAGGGGGATAACTACTGGAAACGGTAGCTAATACCGCATA

ACGTCGCAAGACCAAAGAGGGGGACCTTCGGGCCTCTTGCCATCGGATGT

GCCCAGATGGGATTAGCTAGTAGGTGGGGTAACGGCTCACCTAGGCGACG

ATCCCTAGCTGGTCTGAGAGGATGACCAGCCACACTGGAACTGAGACACG

GTCCAGACTCCTACGGGAGGCAGCAGTGGGGAATATTGCACAATGGGCGC

AAGCCTGATGCAGCCATGCCGCGTGTATGAAGAAGGCCTTCGGGTTGTAA

AGTACTTTCAGCGGGGAGGAAGGGAGTAAAGTTAATACCTTTGCTCATTG

ACGTTACCCGCAGAAGAAGCACCGGCTAACTCCGTGCCAGCAGCCGCGGT

AATACGGAGGGTGCAAGCGTTAATCGGAATTACTGGGCGTAAAGCGCACG

CAGGCGGTTTGTTAAGTCAGATGTGAAATCCCCGGGCTCAACCTGGGAAC

TGCATCTGATACTGGCAAGCTTGAGTCTCGTAGAGGGGGGTAGAATTCCA

GGTGTAGCGGTGAAATGCGTAGAGATCTGGAGGAATACCGGTGGCGAAGG

CGGCCCCCTGGACGAAGACTGACGCTCAGGTGCGAAAGCGTGGGGAGCAA

ACAGGATTAGATACCCTGGTAGTCCACGCCGTAAACGATGTCGACTTGGA

GGTTGTGCCCTTGAGGCGTGGCTTCCGGAGCTAACGCGTTAAGTCGACCG

CCTGGGGAGTACGGCCGCAAGGTTAAAACTCAAATGAATTGACGGGGGCC

CGCACAAGCGGTGGAGCATGTGGTTTAATTCGATGCAACGCGAAGAACCT

TACCTGGTCTTGACATCCACGGAAGTTTTCAGAGATGAGAATGTGCCTTC

GGGAACCGTGAGACAGGTGCTGCATGGCTGTCGTCAGCTCGTGTTGTGAA

ATGTTGGGTTAAGTCCCGCAACGAGCGCAACCCTTATCCTTTGTTGCCAG

CGGTCCGGCCGGGAACTCAAAGGAGACTGCCAGTGATAAACTGGAGGAAG

GTGGGGATGACGTCAAGTCATCATGGCCCTTACGACCAGGGCTACACACG

TGCTACAATGGCGCATACAAAGAGAAGCGACCTCGCGAGAGCAAGCGGAC

CTCATAAAGTGCGTCGTAGTCCGGATTGGAGTCTGCAACTCGACTCCATG

AAGTCGGAATCGCTAGTAATCGTGGATCAGAATGCCACGGTGAATACGTT

CCCGGGCCTTGTACACACCGCCCGTCACACCATGGGAGTGGGTTGCAAAA

GAAGTAGGTAGCTTAACCTTCGGGAGGGCG

**AN-2**

GCAGTCGTACGCACTGGCCCAACTGATTGATGGTGCTTGCACCTGATTGA

CGATGGATCACCAGTGAGTGGCGGACGGGTGAGTAACACGTAGGTAACCT

GCCCCGGAGCGGGGGATAACATTTGGAAACAGATGCTAATACCGCATAAC

AACAAAAGCCGCATGGCTTTTGTTTGAAAGATGGCTTTGGCTATCACTCT

GGGATGGACCTGCGGTGCATTAGCTAGTTGGTAAGGTAACGGCTTACCAA

GGCGATGATGCATAGCCGAGTTGAGAGACTGATCGGCCACAATGGAACTG

AGACACGGTCCATACTCCTACGGGAGGCAGCAGTAGGGAATCTTCCACAA

TGGGCGCAAGCCTGATGGAGCAACACCGCGTGAGTGAAGAAGGGTTTCGG

CTCGTAAAGCTCTGTTGTTGGAGAAGAACGTGCGTGAGAGTAACTGTTCA

CGCAGTGACGGTATCCAACCAGAAAGTCACGGCTAACTACGTGCCAGCAG

CCGCGGTAATACGTAGGTGGCAAGCGTTATCCGGATTTATTGGGCGTAAA

GCGAGCGCAGGCGGTTGCTTAGGTCTGATGTGAAAGCCTTCGGCTTAACC

GAAGAAGTGCATCGGAAACCGGGCAACTTGAGTGCAGAAGAGGACAGTGG

AACTCCATGTGTAGCGGTGGAATGCGTAGATATATGGAAGAACACCAGTG

GCGAAGGCGGCTGTCTGGTCTGCAACTGACGCTGAGGCTCGAAAGCATGG

GTAGCGAACAGGATTAGATACCCTGGTAGTCCATGCCGTAAACGATGAGT

GCTAGGTGTTGGAGGGTTTCCGCCCTTCAGTGCCGGAGCTAACGCATTAA

GCACTCCGCCTGGGGAGTACGACCGCAAGGTTGAAACTCAAAGGAATTGA

CGGGGGCCCGCACAAGCGGTGGAGCATGTGGTTTAATTCGAAGCTACGCG

AAGAACCTTACCAGGTCTTGACATCTTGCGCTAACCTTAGAGATAAGGCG

TTCCCTTCGGGGACGCAATGACAGGTGGTGCATGGTCGTCGTCAGCTCGT

GTCGTGAGATGTTGGGTTAAGTCCCGCAACGAGCGCAACCCTTGTTACTA

GTTGCCAGCATTAAGTTGGGCACTCTAGTGAGACTGCCGGTGACAAACCG

GAGGAAGGTGGGGACGACGTCAGATCATCATGCCCCTTATGACCTGGGCT

ACACACGTGCTACAATGGACGGTACAACGAGTCGCAAGCTCGCGAGAGTA

AGCTAATCTCTTAAAGCCGTTCTCAGTTCGGACTGTAGGCTGCAACTCGC

CTACACGAAGTCGGAATCGCTAGTAATCGCGGATCAGCATGCCGCGGTGA

ATACGTTCCCGGGCCTTGTACACACCGCCCGTCACGCCATGGGAGTTTGT

AACGCCCAAAGTCGGTGGCCTAACCTTTAGGAGGGAGCC

**AN-3**

AGTCGTACGCACTGGCCCAACTGATTGATGGTGCTTGCACCTGATTGACG

ATGGATCACCAGTGAGTGGCGGACGGGTGAGTAACACGTAGGTAACCTGC

CCCGGAGCGGGGGATAACATTTGGAAACAGATGCTAATACCGCATAACAA

CAAAAGCCGCATGGCTTTTGTTTGAAAGATGGCTTTGGCTATCACTCTGG

GATGGACCTGCGGTGCATTAGCTAGTTGGTAAGGTAACGGCTTACCAAGG

CGATGATGCATAGCCGAGTTGAGAGACTGATCGGCCACAATGGAACTGAG

ACACGGTCCATACTCCTACGGGAGGCAGCAGTAGGGAATCTTCCACAATG

GGCGCAAGCCTGATGGAGCAACACCGCGTGAGTGAAGAAGGGTTTCGGCT

CGTAAAGCTCTGTTGTTGGAGAAGAACGTGCGTGAGAGTAACTGTTCACG

CAGTGACGGTATCCAACCAGAAAGTCACGGCTAACTACGTGCCAGCAGCC

GCGGTAATACGTAGGTGGCAAGCGTTATCCGGATTTATTGGGCGTAAAGC

GAGCGCAGGCGGTTGCTTAGGTCTGATGTGAAAGCCTTCGGCTTAACCGA

AGAAGTGCATCGGAAACCGGGCGACTTGAGTGCAGAAGAGGACAGTGGAA

CTCCATGTGTAGCGGTGGAATGCGTAGATATATGGAAGAACACCAGTGGC

GAAGGCGGCTGTCTGGTCTGCAACTGACGCTGAGGCTCGAAAGCATGGGT

AGCGAACAGGATTAGATACCCTGGTAGTCCATGCCGTAAACGATGAGTGC

TAGGTGTTGGAGGGTTTCCGCCCTTCAGTGCCGGAGCTAACGCATTAAGC

ACTCCGCCTGGGGAGTACGACCGCAAGGTTGAAACTCAAAGGAATTGACG

GGGGCCCGCACAAGCGGTGGAGCATGTGGTTTAATTCGAAGCTACGCGAA

GAACCTTACCAGGTCTTGACATCTTGCGCTAACCTTAGAGATAAGGCGTT

CCCTTCGGGGACGCAATGACAGGTGGTGCATGGTCGTCGTCAGCTCGTGT

CGTGAGATGTTGGGTTAAGTCCCGCAACGAGCGCAACCCTTGTTACTAGT

TGCCAGCATTAAGTTGGGCACTCTAGTGAGACTGCCGGTGACAAACCGGA

GGAAGGTGGGGACGACGTCAGATCATCATGCCCCTTATGACCTGGGCTAC

ACACGTGCTACAATGGACGGTACAACGAGTCGCAAGCTCGCGAGAGTAAG

CTAATCTCTTAAAGCCGTTCTCAGTTCGGACTGTAGGCTGCAACTCGCCT

ACACGAAGTCGGAATCGCTAGTAATCGCGGATCAGCATGCCGCGGTGAAT

ACGTTCCCGGGCCTTGTACACACCGCC

**AN-4**

GAGTAATGTCTGGGAAACTGCCTGATGGAGGGGGATAACTACTGGAAACG

GTAGCTAATACCGCATAACGTCGCAAGACCAAAGAGGGGGACCTTCGGGC

CTCTTGCCATCGGATGTGCCCAGATGGGATTAGCTAGTAGGTGGGGTAAC

GGCTCACCTAGGCGACGATCCCTAGCTGGTCTGAGAGGATGACCAGCCAC

ACTGGAACTGAGACACGGTCCAGACTCCTACGGGAGGCAGCAGTGGGGAA

TATTGCACAATGGGCGCAAGCCTGATGCAGCCATGCCGCGTGTATGAAGA

AGGCCTTCGGGTTGTAAAGTACTTTCAGCGGGGAGGAAGGGAGTAAAGTT

AATACCTTTGCTCATTGACGTTACCCGCAGAAGAAGCACCGGCTAACTCC

GTGCCAGCAGCCGCGGTAATACGGAGGGTGCAAGCGTTAATCGGAATTAC

TGGGCGTAAAGCGCACGCAGGCGGTTTGTTAAGTCAGATGTGAAATCCCC

GGGCTCAACCTGGGAACTGCATCTGATACTGGCAAGCTTGAGTCTCGTAG

AGGGGGGTAGAATTCCAGGTGTAGCGGTGAAATGCGTAGAGATCTGGAGG

AATACCGGTGGCGAAGGCGGCCCCCTGGACGAAGACTGACGCTCAGGTGC

GAAAGCGTGGGGAGCAAACAGGATTAGATACCCTGGTAGTCCACGCCGTA

AACGATGTCGACTTGGAGGTTGTGCCCTTGAGGCGTGGCTTCCGGAGCTA

ACGCGTTAAGTCGACCGCCTGGGGAGTACGGCCGCAAGGTTAAAACTCAA

ATGAATTGACGGGGGCCCGCACAAGCGGTGGAGCATGTGGTTTAATTCGA

TGCAACGCGAAGAACCTTACCTGGTCTTGACATCCACGGAAGTTTTCAGA

GATGAGAATGTGCCTTCGGGAACCGTGAGACAGGTGCTGCATGGCTGTCG

TCAGCTCGTGTTGTGAAATGTTGGGTTAAGTCCCGCAACGAGCGCAACCC

TTATCCTTTGTTGCCAGCGGTCCGGCCGGGAACTCAAAGGAGACTGCCAG

TGATAAACTGGAGGAAGGTGGGGATGACGTCAAGTCATCATGGCCCTTAC

GACCAGGGCTACACACGTGCTACAATGGCGCATACAAAGAGAAGCGACCT

CGCGAGAGCAAGCGGACCTCATAAAGTGCGTCGTAGTCCGGATTGGAGTC

TGCAACTCGACTCCATGAAGTCGGAATCGCTAGTAATCGTGGATCAGAAT

GCCACGGTGAATACGTTCCCGGGCCTTGTACACACCGCCCAACACACCAT

GGGAGTGGGTTGCAAAAGAAGTAGGTAGCTTAACCTTCGGGAG
