# Supplementary figures and images for: Mycobacterium tuberculosis Rv1987 protein attenuates inflammatory response and consequently alters microbiota in mouse lung
Source: Front Cell Infect Microbiol. 2023 Nov 1;13:1256866. doi: 10.3389/fcimb.2023.1256866 (PMC10646435; doi:10.3389/fcimb.2023.1256866)

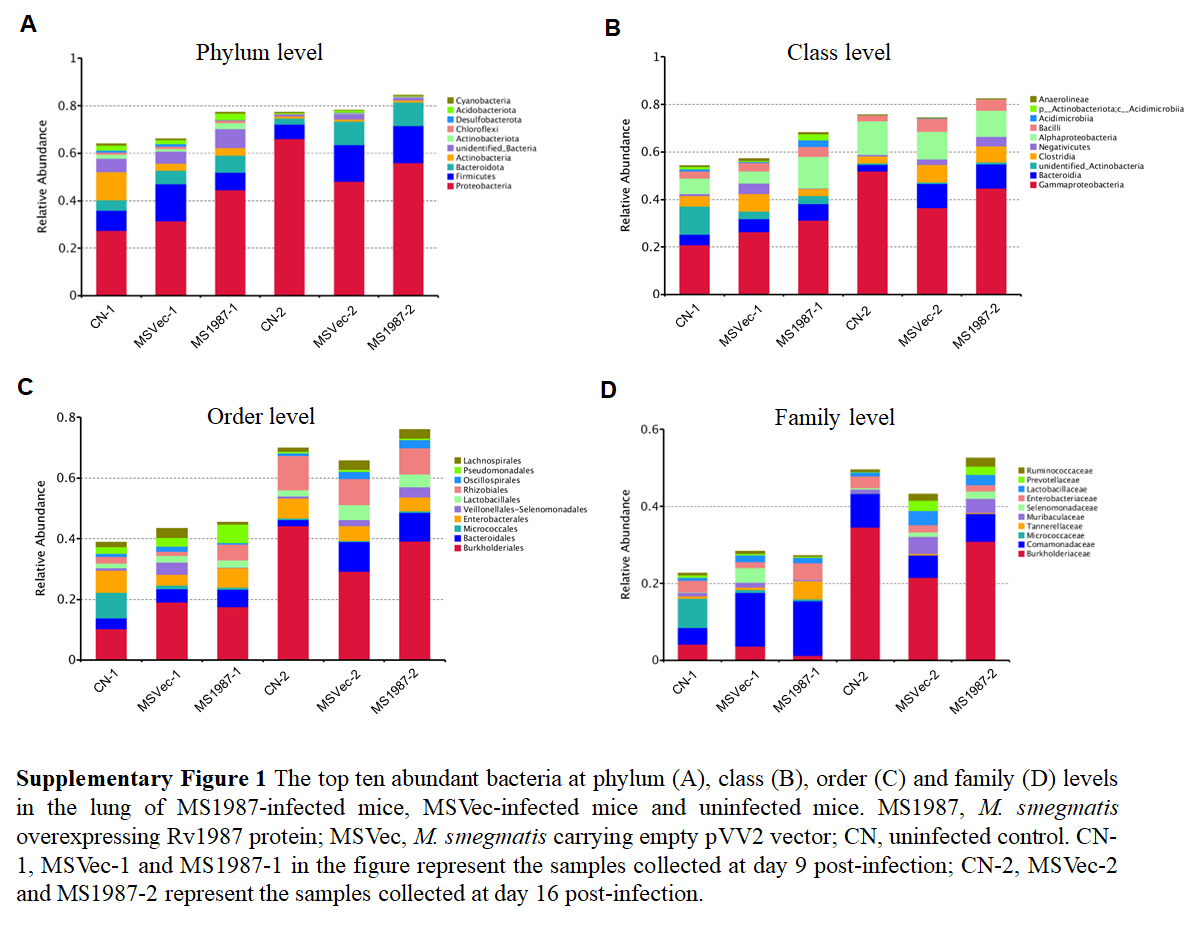

Supplement: Supplementary file 7 [file Image_1.tif]

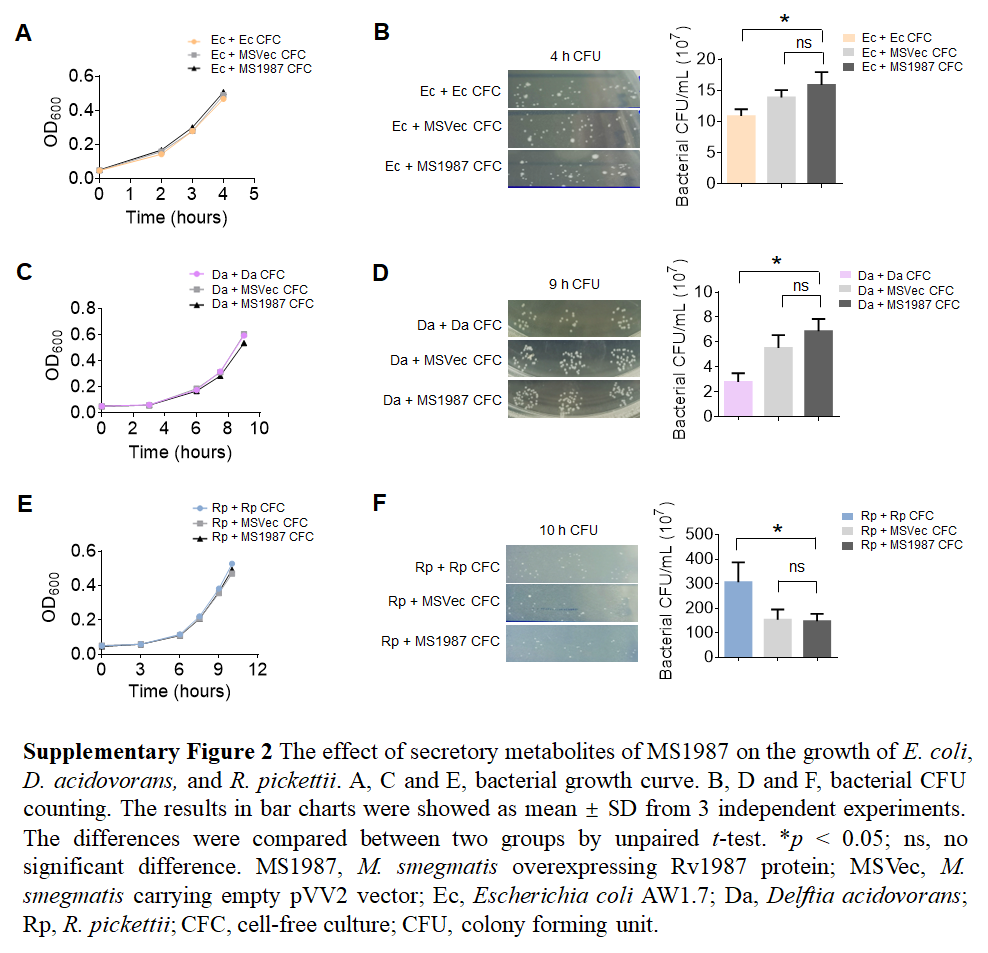

Supplement: Supplementary file 8 [file Image_2.tif]
