# Supplementary material for: Mycobacterium tuberculosis Rv1987 protein attenuates inflammatory response and consequently alters microbiota in mouse lung
Source: Front Cell Infect Microbiol. 2023 Nov 1;13:1256866. doi: 10.3389/fcimb.2023.1256866 (PMC10646435; doi:10.3389/fcimb.2023.1256866)
Supplement: Supplementary file 9 [file Table_1.docx]

Supplementary Table 1. Primers used for qPCR.

| Gene | Source | Primer sequence |
| --- | --- | --- |
| *IL-10* | mouse | 5’ AAGCCTTATCGGAAATGATCCA 3’  5’ GCTCCACTGCCTTGCTCTTATT 3’ |
| *IL-12 p40* | mouse | 5’ GAAGTTCAACATCAAGAGCAGTAG 3’  5’ GGACACTGAATACTTCTCAT 3’ |
| *TNF-α* | mouse | 5’ CAGCCGATTTGCTATCTCATACC 3’  5’ GTACTTGGGCAGATTGACCTCAG 3’ |
| *IL-4* | mouse | 5’ ACAGGAGAAGGGACGCCAT 3’  5’ GAAGCCCTACAGACGAGCTCA 3’ |
| *IL-17* | mouse | 5’ ATCTGTGTCTCTGATGCTGTTGC 3’  5’ CACGCTGAGCTTTGAGGGA 3’ |
| *IFN-γ* | mouse | 5’ TCAAGTGGCATAGATGTGGAAGA 3’  5’ TCAGGTGTGATTCAATGACGCT 3’ |
| *IL-6* | mouse | 5’ GAACAACGATGATGCACTTG 3’  5’ ATGTACTCCAGGTAGCTATG 3’ |
| *β-actin* | mouse | 5’ AGAGGGAAATCGTGCGTGAC 3’  5’ CAATAGTGATGACCTGGCCGT 3’ |
